# Spatial variations of community structures and methane cycling across a transect of Lei-Gong-Hou mud volcanoes in eastern Taiwan

**DOI:** 10.3389/fmicb.2014.00121

**Published:** 2014-03-25

**Authors:** Pei-Ling Wang, Yi-Ping Chiu, Ting-Wen Cheng, Yung-Hsin Chang, Wei-Xain Tu, Li-Hung Lin

**Affiliations:** ^1^Institute of Oceanography, National Taiwan UniversityTaipei, Taiwan; ^2^Department of Geosciences, National Taiwan UniversityTaipei, Taiwan

**Keywords:** mud volcano, methanogenesis, methanotrophy, metal reduction, ANME group, Taiwan

## Abstract

This study analyzed cored sediments retrieved from sites distributed across a transect of the Lei-Gong-Hou mud volcanoes in eastern Taiwan to uncover the spatial distributions of biogeochemical processes and community assemblages involved in methane cycling. The profiles of methane concentration and carbon isotopic composition revealed various orders of the predominance of specific methane-related metabolisms along depth. At a site proximal to the bubbling pool, the methanogenic zone was sandwiched by the anaerobic methanotrophic zones. For two sites distributed toward the topographic depression, the methanogenic zone overlaid the anaerobic methanotrophic zone. The predominance of anaerobic methanotrophy at specific depth intervals is supported by the enhanced copy numbers of the ANME-2a 16S rRNA gene and coincides with high dissolved Fe/Mn concentrations and copy numbers of the *Desulfuromonas*/*Pelobacter* 16S rRNA gene. Assemblages of 16S rRNA and *mcrA* genes revealed that methanogenesis was mediated by *Methanococcoides* and *Methanosarcina*. *pmoA* genes and a few 16S rRNA genes related to aerobic methanotrophs were detected in limited numbers of subsurface samples. While dissolved Fe/Mn signifies the presence of anaerobic metabolisms near the surface, the correlations between geochemical characteristics and gene abundances, and the absence of aerobic methanotrophs in top sediments suggest that anaerobic methanotrophy is potentially dependent on iron/manganese reduction and dominates over aerobic methanotrophy for the removal of methane produced *in situ* or from a deep source. Near-surface methanogenesis contributes to the methane emissions from mud platform. The alternating arrangements of methanogenic and methanotrophic zones at different sites suggest that the interactions between mud deposition, evaporation, oxidation and fluid transport modulate the assemblages of microbial communities and methane cycling in different compartments of terrestrial mud volcanoes.

## Introduction

Terrestrial mud volcanoes are ubiquitous in compressional tectonic regimes (Kopf, 2004). These peculiar cone-shaped features expel fluidized muds or breccia through either seepages or explosive discharges (Mazzini, 2009). Often, methane constitutes the major gaseous phase accompanying the release of fluids and sediments (Dimitrov, 2003). Despite focused flows channeling along the fracture network, deeply-sourced gases and fluids also percolate through pore space of sediments via diffusion and/or advection (Milkov, 2005; Mazzini et al., 2009). Such pervasive gas transport underneath mud volcanoes enables even greater summed methane emissions from surrounding mud platforms (termed micro- or mini-seepage) when compared with those from main conduits or fractures (termed macro-seepage) (Etiope et al., 2004a,b; Hong et al., 2013). Unlike marine counterparts where the overlying seawater can buffer methane released from the sediment-seawater interface (Yvon-Lewis et al., 2011), terrestrial mud volcanoes directly emit methane and gaseous hydrocarbons into the atmosphere. As the methane greenhouse effect is greater than that of CO_2_ by a factor of ~25, terrestrial mud volcanoes are considered a potent contributor intensifying climatic fluctuations on contemporary and geological time scales (Etiope et al., 2008).

The exact magnitude of methane released from or retained within terrestrial mud volcanoes is dependent on several factors. In addition to fluid transport, *in situ* microbial production and consumption appear to be the most critical factor governing methane abundances in the pore space and methane fluxes across the sediment-air interface (Alain et al., 2006; Chang et al., 2012; Cheng et al., 2012). In particular, terrestrial mud volcanoes are generally limited in sulfate. Sulfate-dependent methanotrophy commonly observed in marine counterparts (Knittel and Boetius, 2009) would be likely inhibited under such sulfate-deficient conditions, allowing more methane emitted into the atmosphere. Using geochemical profiles, incubation approaches, and 16S rRNA gene abundances and assemblages, previous studies indicated that methane originating from deep sources mixes with microbial methane produced at shallow depths (Chang et al., 2012; Cheng et al., 2012). The quantities of microbial methane produced *in situ* are two to three times those of deeply-sourced methane, providing abundant electron donors for methanotrophy. In addition, anaerobic methanotrophs (ANME-1 to -3 lineages) proliferate in methane-transition zones, likely being coupled with either sulfate or iron/manganese reduction fueled by mineral-derived sulfate or iron/manganese oxide generated from the atmospheric oxidation of reduced sulfur or metals (Chang et al., 2012; Cheng et al., 2012). Such subsurface-surface interactions create steep redox gradients that lead to the compartmentalization of different metabolic schemes into discrete horizons.

Metabolic stratification driven by subsurface-surface interactions in terrestrial mud volcanoes is persistent over years, effectively removing methane from the pore space (Chang et al., 2012; Cheng et al., 2012). However, these observations are primarily drawn from cores retrieved near fluid conduits. Considering that a typical mud volcano is cone-shaped, methane transport and emission may be susceptible to factors that would vary on multiple dimensions. First, advective methane fluxes originating from deep sources are expected to decrease as the distance from the major conduit or bubbling pool increases (Etiope et al., 2004a,b). The decrease in methane flux also reflects the reduced flux of reducing fluid, thereby favoring the penetration of aerobic methanotrophy and other aerobic metabolisms into deeper levels while inhibiting anaerobic methanotrophy, methanogenesis, and other anaerobic metabolisms. Second, top sediments distributed along the slope of mud volcanoes are episodically immersed by fluids expelled from bubbling pools. Solute contents in pore water could be enhanced due to the loss of pore water through evaporation (Svensen et al., 2007). Previous studies have demonstrated that evaporation could rapidly enhance solute concentrations, even for samples located close to the bubbling pool (Chang et al., 2012; Cheng et al., 2012). Therefore, increasing distance from the bubbling pool is expected to generate enrichments of solutes in the top pore water downslope the cone-shaped structure. Such a salinity increase would pose various effects on community assemblages and metabolic pathways. Determining the spatial variations of pore water geochemistry and microbial community structure would provide a basis for the assessment of total methane emission from a mud volcano.

The aim of this study was to identify the architecture of microbial methane producing and consuming zones in a sulfate-deficient terrestrial mud volcano using geochemical patterns and community assemblages based on 16S rRNA and methane-related functional genes. This study extends previous efforts (Chang et al., 2012) to a broader spatial scale by analyzing sediments across a transect of a cone-shaped feature at the Lei-Gong-Hou mud volcanoes (LGHMVs) in eastern Taiwan. Geochemical profiles, and gene assemblages and abundances were integrated with those obtained previously (Chang et al., 2012) to address the spatial variations in microbial cycling of methane.

## Materials and methods

### Site background and sample acquisition

**Figure 1:**
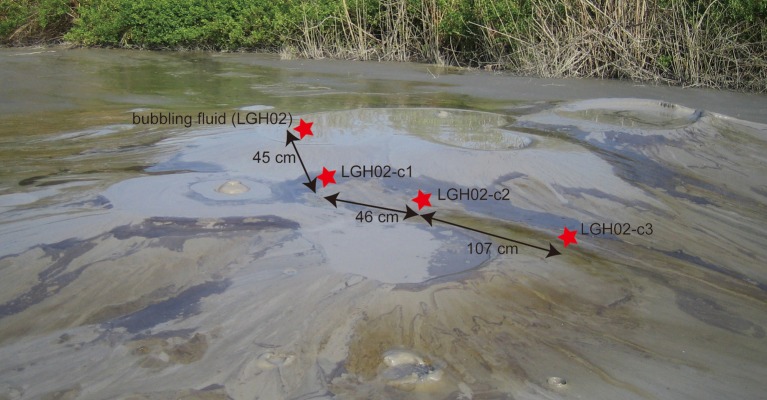
**Field photo of the study sites at the LGHMVs in eastern Taiwan**. The investigated area is located in the topographic high where a cluster of at least six bubbling pools formed. Bubbling fluids from pool LGH02 and three cores located downslope along the gulley between four bubbling pools were collected in 2009. Black oily and yellowish patches were distributed in the mud platform near the bubbling pools and became extinct in the dry area.

Samples were collected from the LGHMVs (22°58'59.02”N, 121°12'33.85”E) in the Kuan-Shan area in eastern Taiwan (Figure 1). The stratum hosting the LGHMVs, the Li-Chi Formation, is distributed trending north-south on the east side of the Longitudinal Valley that separates the Coastal Range to the east from the metamorphic complex to the west (Chang et al., 2001). The Li-Chi Formation is composed of primarily clays mixed with rock fragments in different sizes and lithologies, and represents the intra-arc sediments that formed during the convergence between the Eurasian and Philippine Sea Plates (Chang et al., 2001).

The LGHMVs are composed of more than ten bubbling, cone-shaped features distributed along a nearly north-south transect potentially related to the Longitudinal Fault. Muddy fluids with abundant hydrocarbon gases were discharged in every pool during sampling in February 2009 (Chang et al., 2012). Previous studies indicated that exsolved gases from bubbling pools consist primarily of methane (90–98%) with δ^13^C values ranging between −51 and −49‰ [referenced to the Vienna Pee Dee Belemnite (VPDB)] (Sun et al., 2010).

In addition to the bubbling pool (LGH02-ew) and cored sediments (LGH02-c1) used for the previous study (Chang et al., 2012), two additional cores (LGH02-c2 and -c3) at a distance of 46 and 153 cm from core LGH02-c1 were also collected (Figure 1). These two cores had a total length of 20 cm and were sectioned (mostly at a 2-cm interval) for analyses of gas and aqueous geochemistry, 16S rRNA gene and methane-related functional genes. Samples collected from LGH02-ew and core LGH02-c1 were also used for analyses of methane-related functional genes. Gears for core retrieval and sample processing in the field were heat-sterilized prior to the field trip. All samples were stored either at room temperature (for gas analyses) or on dry ice (for aqueous chemistry and molecular analyses) during transportation, and transferred back to the laboratory within 6 h. The reported depth represents the mean of depth across the section interval.

### Sample processing and analysis in the laboratory

Upon arriving at the laboratory, samples for aqueous geochemistry and DNA analyses were immediately subject to centrifugation at ~8200 ×g for 15 min. Supernatants were decanted from the tubes, filtered using 0.22 μm pore-sized membranes, and split into two fractions; one was treated with 10% by volume of 2 M nitric acid for the preservation of cations, and the other was used for anions without any preservative. One milliliter of the anion sample was fixed with 0.1 mL of 1 M Zn-acetate for the determination of HS^−^ concentration. Geochemical and DNA samples were stored in a 4°C refrigerator and a −80°C freezer, respectively, until further analysis.

The abundances of major anions, dissolved Fe and Mn, sulfide, and methane, and carbon isotopic compositions of methane were measured using the same methods as those described in Chang et al. (2012). The measured partial pressure of methane was converted to the dissolved concentration using the Henry's law constant (Wiesenburg and Guinasso, 1979). The isotopic compositions were reported as δ notation [δ value = (*R*_sample_/*R*_std_-1) × 1000‰, where *R* is the ^13^C/^12^C ratio and std is the VPDB]. The uncertainties for aqueous chemistry and gas abundance were ±2 and ±5%, respectively, whereas the uncertainty for δ^13^C-CH_4_ was ±0.3‰.

Genomic DNA was extracted from 10 g of sediments at 0.5, 2, 4, 6, 11, 15, and 19 cm of core LGH02-c2 and 2, 7, 11, and 19 cm of core LGH02-c3 using the MoBio Ultraclean Mega DNA Prep Soil Kit (MoBio, USA) according to the manufacturer's instruction and stored in water at −20°C. Nearly complete 16S rRNA gene sequences were amplified from the crude extracts from 0.5, 2, 4, 6, and 15 cm of core LGH02-c2 and 2, 11, and 19 cm of core LGH02-c3 using the primer pairs B27f/U1492r for bacteria (Lane, 1991) and A8f/U1513r for archaea (Huber et al., 2002) by polymerase chain reaction (PCR). The amplification scheme was the same as that described in Chang et al. (2012). For the gene *mcrA*, which encodes methyl coenzyme M reductase subunit A (a key enzyme for methanogenesis and anaerobic methanotrophy), only the crude extracts obtained from 3 and 7 cm of core LGH02-c1, 6 and 15 cm of core LGH02-c2, and 2 and 11 cm of core LGH02-c3 were amplified using the primer pair ME1/ME2 (Scholten et al., 2005). Thermal cycling was performed with an initial melting step at 94°C for 2 min, followed by 30 cycles of denaturation at 94°C for 30 s, annealing at 48°C for 45 s, and elongation at 72°C for 1 min, and a final elongation step at 72°C for 7 min. The gene *pmoA*, encoding particulate methane monooxygenase subunit A (a key enzyme for aerobic methane oxidation), was amplified from every crude extract obtained from LGH02-ew and cores LGH02-c1 to -c3 using the primer pair of pmoA189f/pmoA682r or pmoA189f/mb661r (Costello and Lidstrom, 1999). Thermal cycling was performed with an initial melting step at 94°C for 2 min, followed by 40 cycles of denaturation at 94°C for 30 s, annealing at 56°C for 45 s, and elongation at 72°C for 1 min, and a final elongation step at 72°C for 7 min. Three to five positive PCR products were pooled, purified using the StrataPrep PCR purification kit (Stratagene, USA) and cloned using the pGEM-T cloning kit (Promega, USA) following the manufacturers' protocols. Positive inserts from each clone library were sequenced using the primers described above for 16S rRNA genes, and the primer pair M13f/M13r for methane-related functional genes. Parallel negative control was performed to assess the potential contamination introduced during the laboratory manipulation.

The obtained sequences were checked for chimera formation using the Pintail (Ashelford et al., 2005), and aligned to the closely related sequences retrieved from the GenBank and RDP databases using the Greengenes (http://greengenes.lbl.gov) (Desantis et al., 2006). Sequences were categorized into the operational taxonomic units (OTUs) on the basis of 98% similarity cutoff. Taxonomic assignments for representative sequences were based on the results obtained from the SILVA tools (Quast et al., 2013). The UniFrac analyses was based on the phylogenetic trees constructed by the neighbor-joining algorithm and the substitution model of the maximum composite likelihood embedded within the MEGA 4.0 program (Tamura et al., 2007), and employed the environmental cluster algorithm for unweighted OTUs (Lozupone and Knight, 2005). Phylogenetic trees for *pmoA* and *mcrA* genes were constructed with the neighbor-joining algorithm on amino acid sequences deduced from nucleotide sequences using the MEGA 4.0 program. The bootstrap number was computed on the basis of 1000 iterations. The accession numbers of the unique sequences deposited in the GenBank are HQ916477-HQ916659, JN811694-JN811725, JQ407233-JQ407300, JX648556-JX648564, and JX648584- JX648591 for 16S rRNA genes, JX648565-JX648573 for *mcrA* genes, and JX648574-JX648583 for *pmoA* genes.

Quantitative PCR (qPCR) analyses of 16S rRNA gene copy number of bacteria, archaea, ANME-2a and *Desulfuromonas*/*Pelobacter* were performed for every crude extract from cores LGH02-c2 and -c3 on a MyiQ Real Time PCR Detection system (Bio-Rad, USA). In brief, each qPCR reaction contained 1X of SsoFast EvaGreen Supermix (Bio-Rad, USA), 100 nM of each primer, and 2 μ L of template. Primers and PCR conditions were the same as those described in Chang et al. (2012). Primer specificity was first confirmed through sequencing cloned amplicons generated from qPCR assays and performing taxonomic analyses of obtained sequences. For routine qPCR analyses, primer specificity was checked by both melting curve analysis and gel electrophoresis. The annealing was set at the temperature generating the greatest yield [the lowest threshold cycle (C_T_)] while primer specificity was maintained. The standards were prepared from the cloned amplicons of these target groups obtained in the clone libraries. The concentrations of standard DNA were determined using a Qubit spectrophotometer (Invitrogen, USA). The standard DNA templates were then diluted in a 10-fold series and qPCR-amplified in order to measure the C_T_ for a known concentration of standard DNA. The plots of C_T_ vs. standard DNA concentration yielded the linear relationship with *R*^2^ values greater than 0.99. The 16S rRNA gene copy number of a specific group was then calculated assuming 650 g mole^−1^ of one base pair of DNA.

## Results

### Geochemical characteristics

**Figure 2:**
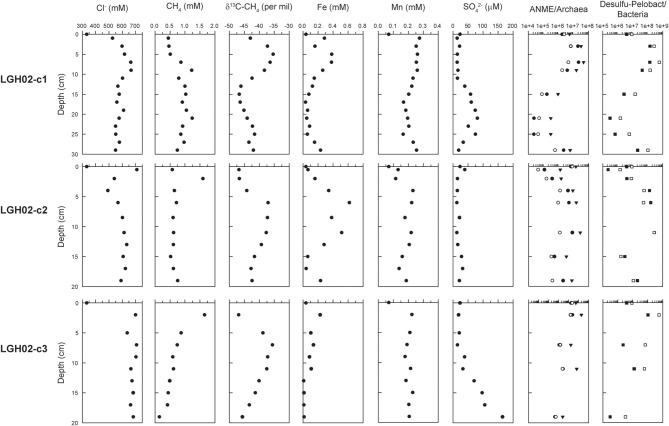
**Pore water geochemistry and 16S rRNA gene abundances along depth**. Target taxonomic units for qPCR analyses include archaea (solid triangles), ANME-2a amplified by primers A426f (solid circles) and A426fm (open circles), bacteria (open squares) and *Desulfuromonas*/*Pelobacter* (solid squares). Data points at 0 cm represent the characteristics of the bubbling fluids. The results obtained from core LGH02-c1 are adopted from Chang et al. (2012).

Analyses of pore water extracted from sediments yielded distinct geochemical characteristics between cores LGH02-c2 and -c3 (Figure 2). Chloride concentrations in core LGH02-c2 decreased from 709 mM at the top to 490 mM at 4 cm, followed by an increase to a level between 567 and 634 mM at depth. In core LGH02-c3, chloride concentrations fluctuated between 637 and 708 mM. Sulfate remained at a low level (<40 μM) in core LGH02-c2, whereas a significant increase from 18 to 165 μM with depth was observed in core LGH02-c3. Dissolved Mn concentrations in core LGH02-c2 increased from 0.13 mM in the top sediments to 0.23 mM at 4 cm and varied between 0.13 and 0.23 mM at depth. In contrast, dissolved Mn concentrations in core LGH02-c3 were at a nearly constant level of 0.20 ± 0.02 mM (average with one standard deviation). Dissolved Fe concentrations in core LGH02-c2 exhibited a peak at 0.62 mM at 6 cm followed by a decrease toward shallower and greater depths. For comparison, dissolved Fe concentrations in core LGH02-c3 decreased from 0.23 to 0.015 mM with depth. Methane concentrations in core LGH02-c2 increased from 0.58 mM at top to 1.6 mM at 2 cm and dropped to a level of 0.52–0.76 mM at depth, whereas methane concentrations in core LGH02-c3 decreased from 1.65 to 0.15 mM with depth. Patterns of carbon isotopic compositions of methane from both cores were in general comparable to each other with ^13^C-enriched methane at 5–11 cm and ^13^C-depleted methane at shallower and greater depths. Sulfide and nitrate concentrations were below the detection limit.

### Assemblages of 16S rRNA genes

PCR amplification yielded 16S rRNA gene amplicons from all crude extracts. A total of 617 bacterial and 525 archaeal 16S rRNA gene sequences were obtained (Supplementary Tables 1, 2). With a 98% similarity cutoff, 107 bacterial and 47 archaeal OTUs were identified. About 46% of bacterial OTUs and 40% of archaeal OTUs appeared to be singletons (appearing only once in any library).

**Figure 3:**
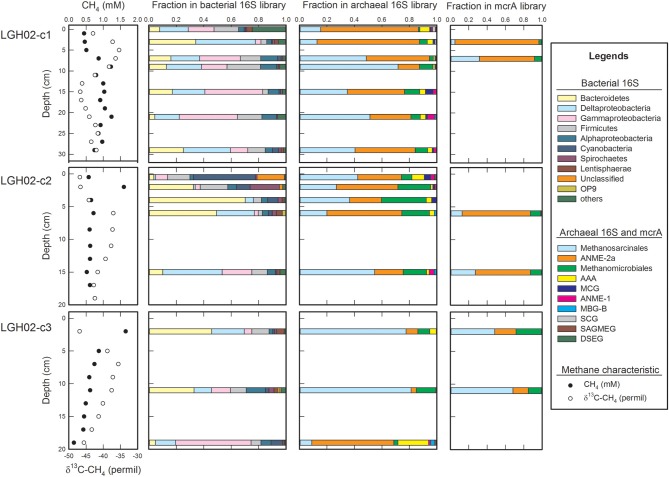
**Community structures along depth on the basis of 16S rRNA and *mcrA* gene sequences**. Profiles of methane concentrations and δ^13^C-CH_4_ values are shown for constraining methanogenic and methanotrophic zones. Abundances of bacterial phyla/divisions less than 1% of the community size are summed and categorized as “others.” The “Methanosarcinales” represents the sequences affiliated with the ANME-2-excluded *Methanosarcinales*. Abbreviations: ANME, ANaerobic Methanotrophic Euryarchaeota; AAA, AOM Associated Archaea; MBG-B, Marine Benthic Group B; SCG, Soil Crenarchaeaota Group; MCG, Miscellaneous Crenarchaeota Group; SAMEG, South Africa Gold Mine Euryarchaeota Group; DSEG, Deep Sea Euryarchaeota Group. Detailed taxonomic categorization and abundances of individual OTUs in individual samples are shown in Supplementary Tables 1, 2.

Bacterial communities were diverse and complexly structured (Figure 3 and Supplementary Table 1). Taxonomic analyses revealed that *Proteobacteria*, *Bacteroidetes*, *Firmicutes*, *Cyanobacteria*, and *Spirochaetes* were the most abundant phyla identified (summed up to be more than 80% of the clones in each clone library). The proportion of each phylum/division varied among the samples. In particular, *Cyanobacteria* outnumbered other phyla/divisions in the top sediments of core LGH02-c2. *Bacteroidetes* constituted the most abundant phylum/division at 2, 4, and 6 cm of core LGH02-c2, and at 2 and 11 cm of core LGH02-c3, whereas *Deltaproteobacteria* and *Gammaproteobacteria* outnumbered others at 15 cm of core LGH02-c2 and 19 cm of core LGH02-c3, respectively. Clone sequences belonging to *Acidobacteria*, *Actinobacteria*, *Chloroflexi*, *Deferribacteres*, *Lentisphaerae*, *Nitrospirae*, OP9, OP11, *Planctomycetes*, *Alphaproteobacteria*, *Betaproteobacteria*, *Epsilonproteobacteria*, and *Zetaproteobacteria* were observed in a minor proportion.

Sequences of dominant bacterial OTUs (>10% of the clones in any individual library) were affiliated with *Bacteroidetes*-related clones recovered from hydrocarbon degradation enrichments (Knight et al., 1999; Alain et al., 2012) (LGH02-B-052 and -053), an uncultured *Cyanobacteria*-related clone from a wetland (LGH02-B-090), uncultured *Deltaproteobacteria*-related clones obtained from the Kazan mud volcano (Pachiadaki et al., 2010) (LGH02-B-027), an uncultured *Desulfuromonadales*-related clone (LGH02-B-030), *Desulfuromonas* spp. (Coates et al., 1995) (LGH02-B-22), an unclassified clone (LGH02-B-178), *Thiohalophilus thiocyanatoxydans* (Sorokin et al., 2007) (LGH02-B-012), and an uncultured *Clostridiales*-related clone from a coral (Sunagawa et al., 2009) (LGH02-B-174). The proportions of these OTUs in each library varied substantially. In particular, OTUs LGH02-B-052 and -053 dominated over others at 2, 4, and 6 cm of core LGH02-c2, and at 2 and 11 cm of core LGH02-c3. OTUs LGH02-B-022 and -030 were abundant at 15 cm of core LGH02-c2, and at 2 and 11 cm of core LGH02-c3. OTUs LGH02-B-090, -174 and -178, and OTU LGH02-B-012 were abundant at shallow intervals of core LGH02-c2 and the deepest interval of core LGH02-c3, respectively. OTU LGH02-B-027 was only detected in core LGH02-c2.

In addition to the major OTUs described above, the OTUs with an overall frequency greater than 1% possessed sequences affiliated with culture sequences belonging to *Desulfovibrio alkaliphilus* (Sorokin et al., 2008) (LGH02-B-136), *Pelobacter acetylenicus* (Schink, 1985) (LGH02-B-024), *Caenispirillum* spp. (LGH02-B-002), *Marinobacter* spp. (Gu et al., 2007) (LGH02-B-011 and -013), *Desulfopila aestuarii* (Suzuki et al., 2007) (LGH02-B-025) and *Spirochaeta* spp. (LGH02-B-150), and with uncultured sequences related to *Bacteroidetes* (Isenbarger et al., 2008) (LGH02-B-054), *Firmicutes* (Hubert et al., 2009; Orcutt et al., 2010) (LGH02-B-066), *Spirochaetes* (LGH02-B-097 and -150), and *Lentisphaerae* (Bano and Hollibaugh, 2002) (LGH02-B-100).

Archaeal communities were essentially composed of ANME members and methanogens although a few crenarchaeal and thaumarchaeal clones were detected (Figure 3 and Supplementary Table 2). Of archaeal OTUs, ANME members constituted 23–55 and 3–60% of clones in individual libraries of cores LGH02-c2 and -c3, respectively. Most detected ANME-related sequences were phylogenetically assigned to the ANME-2a group and shared ≥98% similarity with ANME-2a sequences recovered from core LGH02-c1 (Chang et al., 2012) or 92–97% similarity with sequences from the Nankai Trough (Miyashita et al., 2009), the Baltic Sea (Jagersma et al., 2009), the Okinawa Trough (Inagaki et al., 2006) and other onshore and offshore methane-rich sediments (Hinrichs et al., 1999; Knittel et al., 2005; Alain et al., 2006; Kendall et al., 2007). Few ANME-1 sequences affiliated with those obtained from the Kazan mud volcano (Pachiadaki et al., 2010) and freshwater sediments in the Kanto Plain of Japan (Takeuchi et al., 2009) were recovered from 0.5, 2 and 15 cm of core LGH02-c2 and 19 cm of core LGH02-03. A total of 30 clone sequences from cores LGH02-c2 and -c3 were assigned to the AAA (AOM Associated Archaea) lineage (Supplementary Table 2) that has been proposed to be capable of oxidizing methane anaerobically (Knittel and Boetius, 2009). Most of the AAA-related sequences were clustered into one OTU and particularly enriched at 19 cm of core LGH02-c3 (20% of the clones in the library).

The proportions of *Methanosarcinales*-related clones resembled those of *Methanomicrobiales*-related clones at 2, 4, and 6 cm of core LGH02-c2 (Figure 3). However, *Methanosarcinales*-related clones outnumbered *Methanomicrobiales*-related clones in other samples. The majority of these *Methanosarcinales*-related sequences were affiliated with *Methanosarcina semesiae* (Lyimo et al., 2000, 2009) and *Methanococcoides methylutens* (Sowers and Ferry, 1983), both of which are obligatorily methylotrophic methanogens. Other *Methanosarcinales*-related sequences were affiliated with uncultured sequences recovered from a wetland and from the Kazan mud volcanoes (Pachiadaki et al., 2010), *Methanosaeta* spp. (Zengler et al., 1999), *Methanococcoides* spp. (Asakawa et al., 1998), and *Methanolobus profundi* (Mochimaru et al., 2009). Within *Methanomicrobiales*, sequences related to *Methanoplanus petrolearius* (Ollivier et al., 1997), *Methanocalculus pumilus* (Mori et al., 2000), and uncultured sequences retrieved from the Pearl River estuary (Jiang et al., 2011) were detected. A few sequences affiliated with DSEG and MBG-B from Mediterranean mud volcanoes (Heijs et al., 2007; Kormas et al., 2008) and a hypersaline mat (Robertson et al., 2009), MCG from various environments (Huang et al., 2003; Nelson et al., 2009; Kubo et al., 2012; Lin et al., 2012), SAGMEG from South African gold mine groundwater and a hypersaline mat (Takai et al., 2001; Robertson et al., 2009), and SCG from groundwater in a Japanese gold mine (Nunoura et al., 2005) were also obtained.

### Assemblages of *mcrA* genes

**Figure 4:**
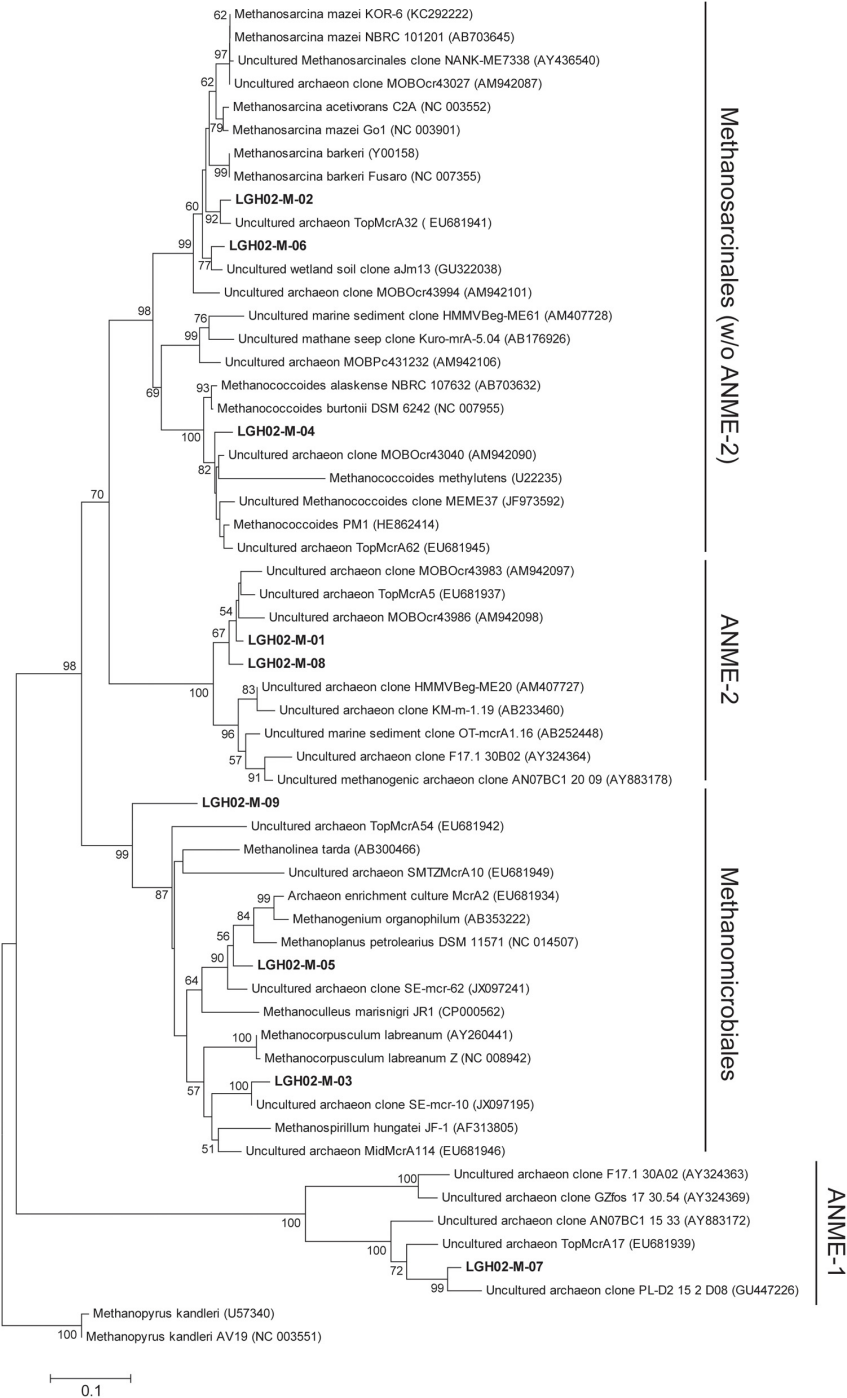
**Phylogenetic tree based on the deduced partial amino acid sequences of *mcrA* genes**. The tree was constructed on the basis of ~263 amino acids. Sequences obtained from this study (in boldface) were categorized into different OTUs on the basis of 98% identity. The scale bar represents the substitution for every base pair. The bootstrap number is shown where greater than 50%.

PCR amplification yielded amplicons of *mcrA* genes for two samples selected from each core. A total of 356 sequences were clustered into nine OTUs (98% similarity) (Figures 3, 4). About 92 and 68% of *mcrA* gene sequences from cores LGH02-c1 and -c2, respectively, were represented by OTU LGH02-M-01 and affiliated with the ANME-2 related sequences recovered from coastal (Roussel et al., 2009) and estuarine sediments (Jiang et al., 2011). In contrast, only 20% of *mcrA* gene sequences from core LGH02-c3 were related to the ANME-2 lineage. The second largest OTU (LGH02-M-02) constituted about 20% of the total clones and possessed sequences affiliated with environmental sequences recovered from estuarine sediments (Jiang et al., 2011) and *Methanosarcina* spp. known to being capable of using acetate and methyl-compounds for methane production (Whitman et al., 2006). About 3–10% of sequences were related to clone sequences from estuarine sediments (Jiang et al., 2011; Xie et al., 2013) and classified into lineages belonging to *Methanospirillum* (LGH02-M-03) *Methanococcoides* (LGH02-M-04), *Methanoplanus* (LGH02-M-05) and *Methanosarcina* (LGH02-M-06). A few sequences were related to the ANME-1 (LGH02-M-07) and *Methanomicrobiales* (LGH-M-09).

### Assemblages of *pmoA* genes

**Figure 5:**
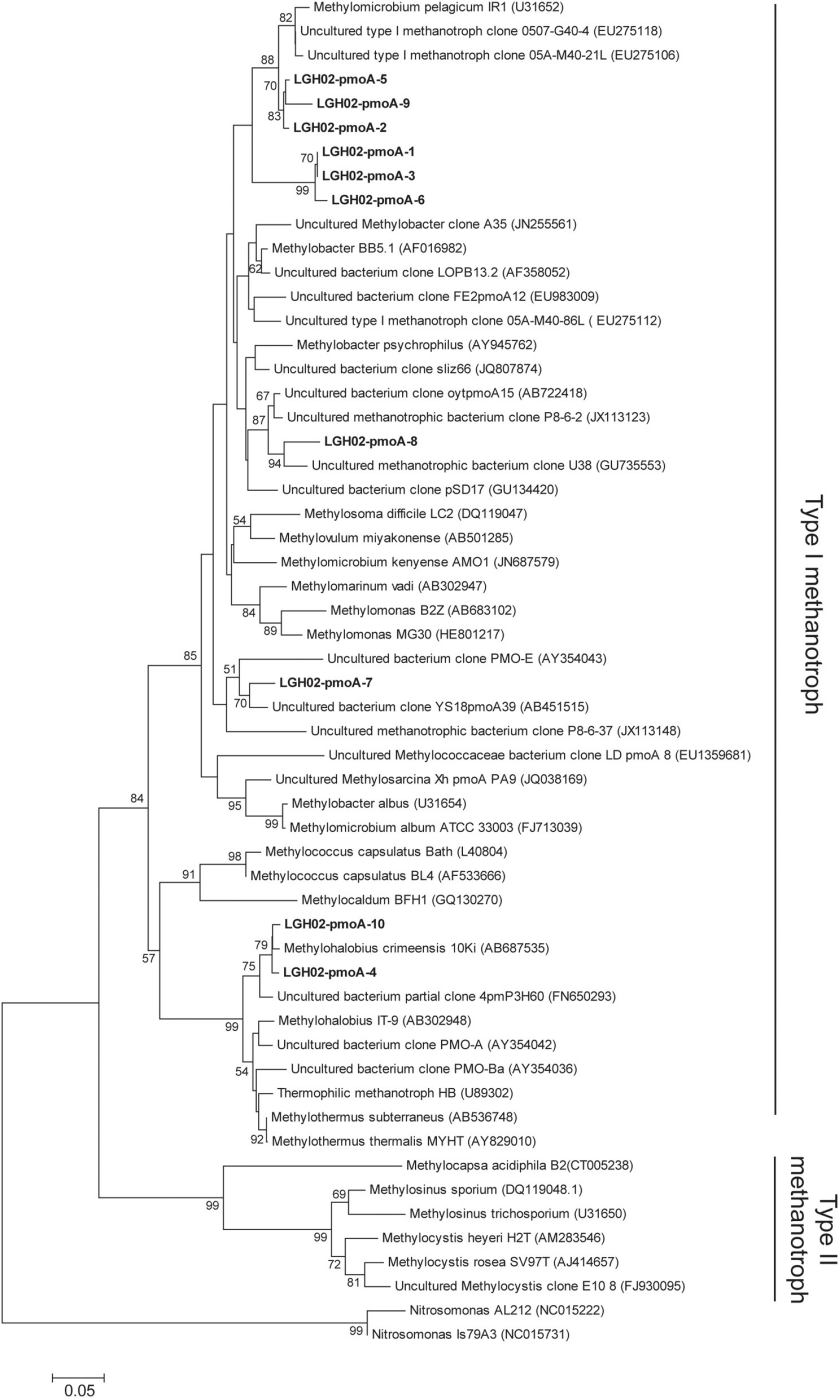
**Phylogenetic tree based on the deduced partial amino acid sequences of *pmoA* genes**. The tree was constructed on the basis of ~152 amino acids. Sequences obtained from this study (in boldface) were categorized into different OTUs on the basis of 98% identity. The scale bar represents the substitution for every base pair. The bootstrap number is shown where greater than 50%.

PCR amplification using the primer pair A189f/A682r did not yield any amplicon. Instead, *pmoA* genes were only detected from sediments at 4, 6, and 11 cm of core LGH02-c2 using the primer pair A189f/mb661. A total of 74 sequences were categorized into ten OTUs at a 98% similarity cutoff (Figure 5). These sequences were classified as type I methanotrophs within the *Gammaproteobacteria*. The majority of these sequences (~64% of the total clones) represented by OTUs LGH02-pmoA-1, −3, and −6 formed a monophyletic lineage that is distinct from all the other reported *pmoA* sequences. The exact topology between this lineage and other methanotrophs or environmental sequences could not be constrained confidently as the bootstrap value was less than 50% on both the amino acid (Figure 5) and nucleic acid levels (data not shown). Comparisons with sequences deposited in the GenBank showed that these sequences were related to uncultured *Methylococcales*-related clones obtained from lake sediments (Lin et al., 2004) or to strain M200 (Kip et al., 2011), *Methylosoma* spp. (Bussmann et al., 2006) and *Methylovurum* spp. (Iguchi et al., 2011), all of which rely on methane and methanol as carbon and energy sources. The other numerically abundant sequences represented by OTUs LGH02-pmoA-2, −5, and −9 were related to *Methylomicrobium pelagicum* (Sieburth et al., 1987; Bowman et al., 1995) or uncultured *Methylococcales*-related clones obtained from landfill soils (Chang et al., 2010). This closest strain also relies on methane and methanol as carbon and energy sources (Sieburth et al., 1987). The proportions of the largest two clusters did not vary systematically with depth. A few sequences were affiliated with *Methylohalobius* spp. isolated from an alkaline lake (Heyer et al., 2005) or with uncultured clones recovered from a hydrothermal field (Kato et al., 2009).

### Abundances of 16S rRNA genes

The qPCR analyses revealed that the gene abundances of all investigated taxonomic groups varied in accordance with the dissolved Fe profiles (Figure 2). The bacterial 16S rRNA gene abundances in core LGH02-c2 ranged between 1.46 × 10^6^ and 2.70 × 10^8^ copies (g^−1^ sediments). Along the depth profile, an increase from 1.46 × 10^6^ to 2.70 × 10^8^ copies (g^−1^ sediments) at ≤11 cm was followed by a decrease of one to two orders of magnitude at 11–19 cm. The *Desulfuromonas*/*Pelobacter* members made up 25 to >100% of the bacterial abundances. Their abundances varied in a fashion similar to those of bacteria. The archaeal 16S rRNA gene abundances ranged between 1.48 × 10^5^ and 1.49 × 10^7^ copies (g^−1^ sediments), whereas the ANME-2a 16S rRNA gene abundances detected by two different ANME-2a specific primers varied from 3.80 × 10^3^ to 2.39 × 10^6^copies (g^−1^ sediments) and from 9.02 × 10^1^ to 1.34 × 10^4^ copies (g^−1^ sediments), respectively. The sum of these two ANME-2a subgroups constituted 3–39% of the archaeal abundances. The gene abundance patterns for archaea and ANME-2a were comparable with those for bacteria and *Desulfuromonas*/*Pelobacter*. In core LGH02-c3, the bacterial 16S rRNA gene abundances decreased from 1.05 × 10^8^ copies (g^−1^ sediments) at 2 cm to 2.29 × 10^6^ copies (g^−1^ sediments) at 7 cm, increased to 1.26 × 10^7^ copies (g^−1^ sediments) at 11 cm, and decreased to 3.10 × 10^5^ copies (g^−1^ sediments) at 19 cm. The abundances of the other investigated taxonomic groups exhibited a pattern similar to that of bacteria.

### Principle component analyses

**Figure 6:**
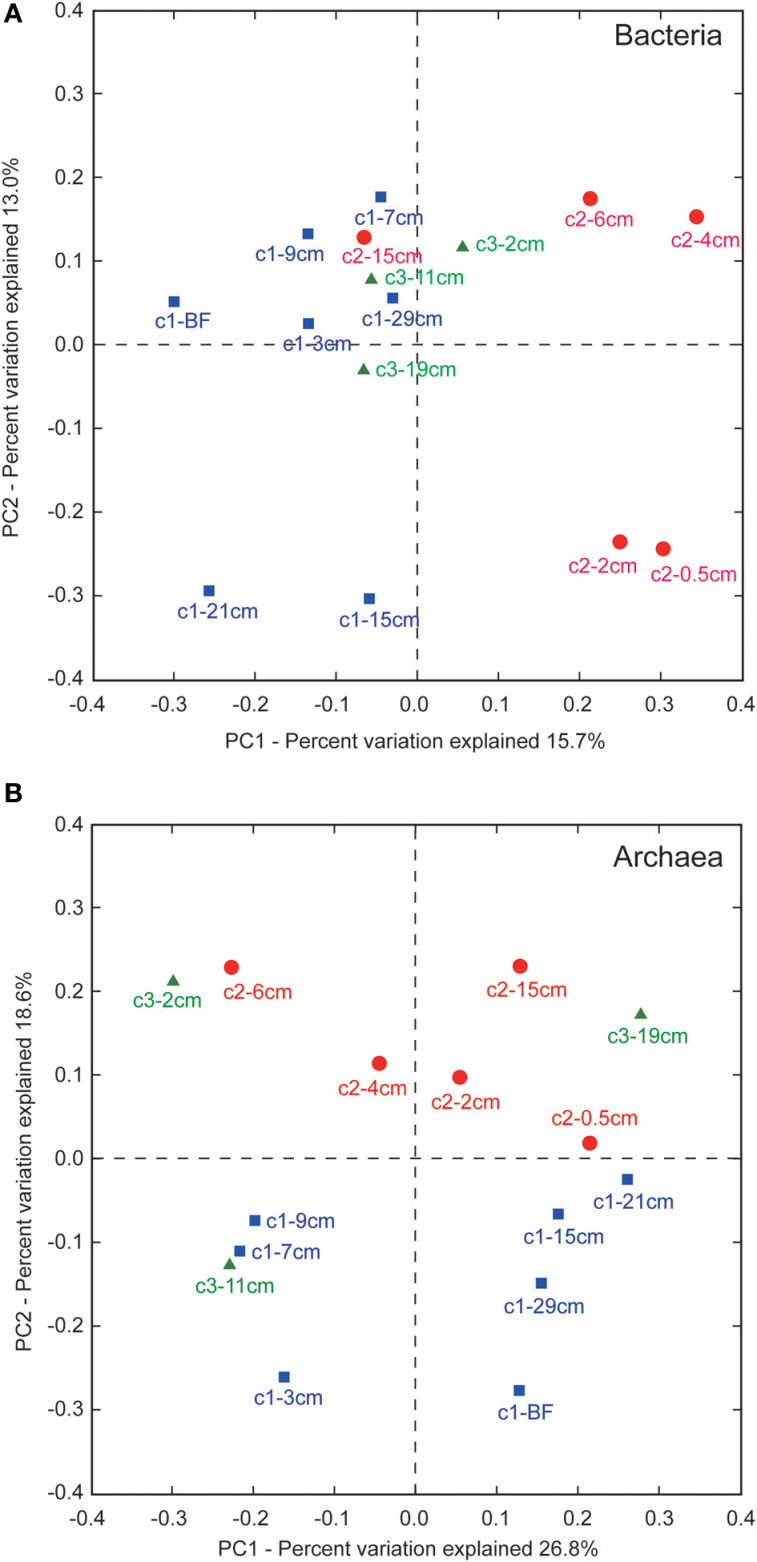
**Unweighted principle component analyses for (A) bacterial and (B) archaeal community assemblages**. Labels for data points consist of core name (c1–c3) and average depth interval. “BF” stands for bubbling fluids.

Principle component analyses (PCA) revealed that PC1 and PC2 could explain ~29 and ~45% the variance in the bacterial and archaeal communities, respectively (Figure 6). For bacteria, communities at 3, 7, 9, and 29 cm of core LGH02-c1, 15 cm of core LGH02-c2, and 2, 11, and 19 cm of core LGH02-c3 clustered near the origin of the plot (Figure 6A). Three other clusters were formed by two samples from similar depths of the same core (groups composed of communities at 15 and 21 cm of core LGH02-c1, of communities at 0.5 and 2 cm of core LGH02-c2, and of communities at 4 and 6 cm of core LGH02-c2). The community associated with the bubbling fluid was distinguished from the groups described above. For archaea, communities at 3, 7, and 9 cm of core LGH02-c1 and 11 cm of core LGH02-c3 formed a cluster in the third quadrant of the plot, whereas communities at 15, 21, and 29 cm of core LGH02-c1 and from the bubbling fluids clustered in the fourth quadrant (Figure 6B). Communities in core LGH02-c2 and at the top and bottom of core LGH02-c3 were distributed over the first and second quadrants.

## Discussion

### Surface and fluid processes

Previous studies have suggested that subsurface-surface interactions are essential to charge the deeply-sourced fluids with substantial amounts of electron acceptors for anaerobic metabolisms in terrestrial mud volcanoes (Chang et al., 2012; Cheng et al., 2012). To assess the role of surface processes in shaping community assemblages at different sites of the LGHMVs, the field observations were examined. The sites for cores LGH02-c1, -c2, and -c3 were distributed along a gulley between four cone-shaped structures covered by patchy yellowish and oily materials (Figure 1). As evidenced by the mud hardness and the distribution of mud crack, the site proximal to the bubbling pool (e.g., core LGH02-c1) was immersed by the expelled fluids at a frequency much greater than the distant site (e.g., core LGH02-c3). The presence of a thick soft mud pile and simultaneous release of mud with fluids also suggest high rates of mud accumulation at sites near the bubbling pool. Therefore, the site for core LGH02-c1 would experience cycled wetting and drying as well as mud deposition at a frequency greater than the other two sites. In contrast, the site for core LGH02-c3 would experience less frequent fluid immersion and mud accumulation than the other two sites, and be subject to more intensive drying and evaporation.

The progressive enhancement of evaporation with increasing distance from the bubbling pool is supported by chloride concentrations in pore water extracted from the cored sediments. When compared with that in the bubbling fluid (333 mM), the chloride concentrations in the top pore water increased from 527 mM for core LGH02-c1 to ~700 mM for the other two cores (Figure 2). The averaged chloride concentration (± one standard deviation) in pore water also increased from 586 ± 41 mM for core LGH02-c1, to 598 ± 58 mM for core LGH02-c2, and 679 ± 23 mM for core LGH02-c3. Considering that the detected chloride concentrations are far less than the halite solubility at the *in situ* temperature, the enhancement in the chloride concentration of the top pore water reflects the effects of short-term evaporation on the overflowing bubbling fluid. Compounds susceptible to microbial transformation (e.g., methane) exhibit drastically different patterns and variation magnitudes from those (e.g., chloride) inert to abiotic and biotic reactions (Figure 2). Such a difference is essentially accounted for by physiological capabilities and activities that would transiently surpass the effect of fluid processes as well as mineral formation, dissolution and sorption (Chang et al., 2012; Cheng et al., 2012). Therefore, potential microbial metabolisms could be determined using the observed geochemical abundances while excluding the signals inherited from abiotic processes.

### Microbial methane cycling

Metabolisms catalyzing methane cycling in the cored sediments were first assessed using the geochemical characteristics of the pore water (Figure 2). Like those interpreted in the previous study (Chang et al., 2012), the variations in concentration and δ^13^C value of methane for core LGH02-c2 suggest the compartmentalization of methanogenesis at <4 cm and anaerobic oxidation of methane (AOM) at 4–13 cm. Aerobic methanotrophy is likely not significant at shallow depths. This is because culture experiments have shown that aerobic methanotrophy preferentially utilizes ^12^CH_4_ over ^13^CH_4_ with an isotopic fractionation magnitude of 2–35‰ (Templeton et al., 2006). If aerobic methane oxidizers are active in consuming methane, the δ^13^C-CH_4_ values would have been shifted toward a higher range. The virtually identical δ^13^C-CH_4_ values at the two shallowest intervals do not corroborate this assertion. In addition, the high concentration of ^13^C-depleted methane and the presence of dissolved Fe/Mn at shallower depths signify methanogenesis and metal reduction, both of which are obligatorily anaerobic metabolisms. The most plausible explanation for the depletion in abundance and isotopic composition near the surface would be the loss of subsurface methane to the atmosphere when taking into account that methane levels in the atmosphere are low (~2 ppmv) and molecular diffusion at the centimeter scale would not cause significant isotopic fractionation. The possibility that aerobic methanotrophy is confined within 0.5 cm could not be completely excluded. Determination of the fine-scaled variations in dissolved methane and oxygen concentration is warranted to address the exact distribution of aerobic methanotrophy.

In addition to the depletion of ^13^C-enriched methane, the AOM zone is also characterized by the enhancement of dissolved Fe and Mn concentrations (up to 0.62 mM Fe and 0.23 mM Mn) (Figure 2). Therefore, high correlations between δ^13^C-CH_4_ values and dissolved Fe/Mn concentrations [Pearson correlation coefficients are 0.73 for Fe and 0.66 for Mn (*p* < 0.05)] are consistent with the presence of metal-dependent anaerobic methanotrophy (Beal et al., 2009; Sivan et al., 2011; Chang et al., 2012). At depths greater than 13 cm of core LGH02-c2, δ^13^C-CH_4_ values decreased to -42‰ while methane concentrations remained at a nearly constant level of 0.6 mM. The depletion of ^13^C in methane was less significant at >13 cm than that at shallow intervals. This combined with a nearly constant concentration of methane suggests that AOM prevails to this depth interval and is sustained by *in situ* methanogenesis and/or deeply-sourced methane, with δ^13^C-CH_4_ values ranging between -51 and -49‰ (Sun et al., 2010).

The profile of methane concentration in core LGH02-c3 was different from that in core LGH02-c2 (Figure 2). While methane concentrations decreased gradually with depth, δ^13^C-CH_4_ values were at a maximum of −36‰ at 7 cm and decreased toward shallower and deeper regions. The geochemical pattern suggests that methanogenesis and AOM were active at <5 and 5–11 cm, respectively. For intervals deeper than 11 cm, the depletion in abundance and isotopic composition of methane contradicts the expression of methanogenesis. Instead, the mixing between residual methane derived from the methanotrophic zone at 5–11 cm and a deep component characterized by the depleted isotopic composition (between −51 and −49‰; Sun et al., 2010) could account for the observed variations. With the exclusion of the data from the depth of 2 cm, δ^13^C-CH_4_ values are correlated with dissolved Fe and sulfate concentrations, respectively [Pearson correlation coefficients are 0.84 for Fe and −0.96 for sulfate (*p* < 0.05)] but irrelevant with dissolved Mn concentrations (*p* > 0.05). These lines of evidence suggest that AOM is potentially dependent on iron and sulfate reduction processes and manganese reduction is driven by organic mineralization.

The geochemical inference for methanotrophy and methanogenesis is supported by the molecular results (Figures 2, 3). Like those observed for core LGH02-c1 (Chang et al., 2012), the gene copy numbers of ANME-2a and *Desulfuromonas/Pelobacter* populations were high at 4–11 cm of core LGH02-c2 (constituting 17–38% of the archaeal populations and >100% of the bacterial populations, respectively). The log-scaled ANME-2a abundances are correlated with the δ^13^C-CH_4_ values [Pearson correlation coefficient is 0.78 (*p* < 0.05)], a pattern consistent with the geochemical interpretation that AOM is active in the methane transition zone. Furthermore, since *Desulfuromonas*/*Pelobacter* members are potential iron/manganese reducers (Lovley, 2006), positive correlation between the gene abundances of *Desulfuromonas*/*Pelobacter* and ANME-2a lineages [Pearson correlation coefficient is 0.99 (*p* < 0.05)] again suggests a likely metabolic interdependence between AOM and iron/manganese reduction. The ANME-2a abundances in core LGH02-c3 exhibited a pattern different from those in the other two cores. The gene copy numbers generally decreased with depth, with an exception that an increase by a factor of ~2 was observed for samples at 7 and 11 cm of core LGH02-c3. The summed ANME-2a abundance at 11 cm (5.24 × 10^5^ copies g^−1^ sediments) was, however, 4–40 times less than those in the methane transition zones of the other two cores. While the high δ^13^C-CH_4_ values indicate the presence of AOM, the low abundance of ANME-2a members together with limited methane depletion at ≥7 cm suggests that AOM might not be as active as in the other two cores.

Methanogens represent the other major component of archaeal communities at the LGHMVs (Figure 3). Their relative abundances in the 16S rRNA or *mcrA* gene clone libraries were inversely correlated with those of the ANME-2a members. Most of these methanogen-related sequences were affiliated with obligatorily methylotrophic methanogens (*Methanococcoides* spp. and *Methanosarcina* spp.; Figures 3, 4) (Asakawa et al., 1998; Lyimo et al., 2000, 2009). These methanogens can proliferate under saline or hypersaline conditions by metabolizing methyl-compounds produced from the fermentation of osmoprotectants (e.g., choline, glycine betaine) that are used to counterbalance osmotic stress (Whitman et al., 2006). The prevalence of *Methanosarcina* and *Methanococcoides* over other methanogens has been constantly observed in hypersaline environments, such as microbial mats in Baja California (Orphan et al., 2008; Smith et al., 2008). However, surface evaporation at the LGHMVs does not generate salinities falling into the hypersaline range. In addition, neither the proportions nor the abundances of these methanogens were correlated with the variations in pore water salinity (*p* > 0.05). As stated previously, the high methane concentrations and low δ^13^C-CH_4_ values near the surface in cores LGH02-c2 and -c3 suggest active methanogenesis. Therefore, the prevalence of these obligatorily methylotrophic methanogens near the surface seems to be best explained by their physiological durability against transient desiccation and oxygen perfusion associated with surface evaporation.

The *pmoA* gene, which is responsible for aerobic methane oxidation, was only detected at 4, 6, and 11 cm of core LGH02-c2. The detected *pmoA* gene sequences were related to type I methanotrophs belonging to the *Gammaproteobacteria* (Figure 5). Most of these sequences were clustered into two OTUs (90% of the total clones) and affiliated with aerobic methanotrophs relying solely on methane and methanol (Sieburth et al., 1987; Bussmann et al., 2006; Iguchi et al., 2011). The presence of these sequences is partly consistent with abundant methane but does not corroborate with the anoxic conditions at these depth intervals geochemically and molecularly interpreted as the AOM zone. Since no culture evidence has attested that these *Gammaproteobacteria* could use nitrate, nitrite, or metal oxides as electron acceptors for methanotrophy, these sequences probably represent the relics of aerobic methanotrophs that proliferate upon exposure to the atmosphere and survive being buried in the anoxic environment. The rare detection of 16S rRNA genes affiliated with known aerobic methanotrophs (7 out of 1305 sequences in Supplementary Table 1) from all three cores and the absence of these sequences in the top sediments further suggest that aerobic methanotrophy might not contribute significantly to the overall methane cycling at the LGHMVs.

Combining geochemical and molecular data reported in this and previous studies (Chang et al., 2012) reveals that methanogenesis and AOM are prevalent regardless of the investigated site. However, their distribution along depth varied from site to site. At the site proximal to the bubbling pool (LGH02-c1), the AOM zone was underlain by the methanogenic and another AOM zones. In contrast, the methanogenic zone overlaid the AOM zone in two other cores (LGH02-c2 and -c3) distributed away from the bubbling pool. The reversible and alternating order of AOM and methanogenic zones along depth is not uncommon at the LGHMVs. For example, the AOM zone was either sandwiched or underlain by the methanogenic zone in other cores retrieved 18 months later from the other sites near bubbling pools (Chang et al., 2012). The alternating distribution of AOM and methanogenic zones is, however, not consistent with the successive predominance of sulfate reduction, AOM, and methanogenesis in marine sediments or terrestrial aquifers, a metabolic zonation pattern controlled by the competition for limited organic matters and H_2_ (Hoehler et al., 1998). Instead, the pattern seems to reflect the development and superposition of one specific metabolic zone over one developed previously, a scenario analogous to mud deposition over time.

The enhanced methane concentrations, low δ^13^C-CH_4_ values, and predominance of sequences affiliated with obligatorily methylotrophic methanogens near the surface also suggest that the mud platform could be an effective source of methane emission. Oxygen penetration is apparently attenuated by active oxygen consumption through biological respiration, and is thereby limited at very shallow intervals. Therefore, oxygen-sensitive methanogenesis could occur close to the surface. Previous studies have revealed that the summed flux of methane emission from the mud platform could exceed that from the corresponding macro-seepages by up to two orders of magnitude and attributed this to the gas transport through either pore space or micro-/mini-seepages (Etiope et al., 2004a,b; Hong et al., 2013). The results observed in this study provide an alternative mechanistic explanation that methanogenesis in near-surface environments can account for enhanced methane emissions from the mud platform.

### Sulfur cycling and other metabolisms

Sulfate concentrations increased from nearly below the limit of detection at 2 cm to 165 μM at the deepest interval in core LGH02-c3 (Figure 2). In addition, sulfate depletion occurred at depth intervals (5–10 cm) coinciding with the high δ^13^C-CH_4_ values, the decrease in methane concentration, and the high dissolved Fe concentrations. Considering that the bubbling fluid with 23 μM sulfate represents a deeply-sourced fluid (Chang et al., 2012), the high sulfate level at depth would require an additional source and could be accounted for by anaerobic sulfur disproportionation or sulfur oxidation potentially driven by the reduction of iron oxyhydroxide (Holmkvist et al., 2011). Such geochemical characteristics also indicate that AOM might be dependent on iron and sulfate reduction.

Analyses of 16S rRNA gene libraries revealed that a great proportion of bacterial sequences at 15 and 21 cm of core LGH02-c1 (18.4 and 8.0%) and 19 cm of core LGH02-c3 (29.3%) were affiliated with sequences retrieved from bubbling fluids and surface sediments of terrestrial and marine mud volcanoes (Yakimov et al., 2002; Omoregie et al., 2008, 2009), and with *Thiohalophilus thiocyanatoxydans* strain HRhD 2 (Sorokin et al., 2007) at ≥96% similarity (Supplementary Table 1). This strain is known to grow using thiosulfate and nitrate as energy sources under saline conditions (1–4 M NaCl) (Sorokin et al., 2007). As sulfate represents the end product from the oxidation of thiosulfate, the observed increase in the sulfate concentration is likely accounted for by the *Thiohalophilus*-related microorganisms. The clone library analyses also indicated that some bacterial sequences were related to *Desulfovibrio alkaliphilus*, *Desulfobacterium* spp. and unclassified *Desulfobacterales* and *Desulfarculales* sequences (Supplementary Table 1). Although the abundances of these potential sulfate reducers were low at these or adjacent intervals, their presence is presumably linked to the observed decrease in sulfate concentration. Whether these potential sulfate reducers are involved in the AOM remains to be investigated.

OTUs LGH02-B-052 and -53 with sequences classified into *Bacteroidetes* were represented by 17.7% of the total clones (Supplementary Table 1). Their proportions in clone libraries were enhanced at depth intervals geochemically and molecularly interpreted as the AOM zone (Figure 2). Most related sequences are derived from mixed populations of polluted sediments and enrichment cultures capable of anaerobically degrading complex organic compounds (e.g., benzene, phenol) (Knight et al., 1999; Alain et al., 2012). Whether and how microorganisms represented by the detected sequences are capable of degrading organic compounds or involved in the metabolic network related to the AOM warrants more investigation. Finally, OTU LGH02-B-90, represented by sequences related to *Cyanobacteria*, constituted 45.0% of the clones recovered from 0.5 cm of core LGH02-c2. Its relative abundance in this core decreased significantly with depth (from 45.0% at 0.5 cm to 1.1% at 15 cm). The decreasing trend of cyanobacteria is consistent with the phototrophic and oxygen producing natures of cyanobacteria, and further suggests that the proliferation of cyanobacteria in the top sediments would intensify the redox gradient and provide an additional pathway of primary production and carbon sequestration in terrestrial mud volcanoes.

### Community patterns

The unweighted Unifrac PCA revealed that bacterial communities in a specific metabolic zone of a core were more similar to each other than those in a different metabolic zone (Figure 6A). For example, samples from 3, 7, 9, and 29 cm and from 15 to 21 cm of core LGH02-c1 were geochemically and molecularly interpreted as the AOM and methanogenic zones, respectively. Communities in these samples were categorized into two clusters in accordance with the interpretations. A similar clustering pattern was observed for core LGH02-c2 (0.5 and 2 cm for methanogenesis, and 4 and 6 cm for AOM) but not for core LGH02-c3. If all communities are pooled together, communities recovered from individual metabolic zones in different cores are not coherently clustered. For archaea, communities from either core LGH02-c1 or -c2 could be differentiated from each other mostly by PC1 (Figure 6B). Furthermore, communities from core LGH02-c1 could be differentiated from those from core LGH02-c2 mostly by PC2. Archaeal communities from core LGH02-c3 were distributed over the entire plot. Compared to bacterial communities, archaeal communities exhibited no systematic variation with respect to depth or metabolic characteristics.

The clustering pattern for bacterial communities from cores LGH02-c1 and -c2 suggests that a specific community function could be mediated by different community members. As the imposed geochemical context varies from site to site, different community members possessing similar metabolic capabilities might be stimulated or inhibited by varying degrees. Therefore, their role in the metabolic network could be possibly swapped among different members whose physiological limitations could be accommodated under a specific geochemical context. In contrast, the lack of correlation between the archaeal assemblage and specific methane-related metabolism suggests that factors other than the observed geochemical characteristics are required to account for community variance. Since most detected archaeal members are affiliated with ANME-2a and *Methanosarcinales*, the community variance could be controlled by the presence or absence of minor community members. Overall, the discordance between community clustering and metabolic interpretation might reflect the heterogeneous nature of geochemical characteristics over various spatial scales in terrestrial mud volcanoes. We note that the community pattern could be biased by PCR or sequencing depth and DNA signatures may not be necessarily translated into metabolic activity or population size.

## Conclusions

The geochemical patterns and assemblages of 16S rRNA and methane-related genes suggest the prevalence and stratified distribution of AOM and methanogenesis at different sites of the LGHMVs. The order of predominant methane-related metabolisms along depth, however, varied from site to site. At the site near the fringe of the bubbling pool, the AOM zone overlaid the methanogenic zone, providing an effective biological filtration for the removal of methane produced *in situ* or migrating from a deep source. For sites away from the bubbling pool, the methanogenic zone overlaid the AOM zone, highlighting a potential contribution of near-surface methanogenesis from the mud platform to the overall methane emission in the area. *In situ* methanogenesis catalyzed by methylotrophic methanogens produced methane at quantities sufficient to fuel anaerobic methanotrophy. Finally, the downcore alternating sequences of the methanogenic and AOM zones at different sites provide evidence that the pattern of metabolic and geochemical zonation is modulated by the frequency and magnitude of mud deposition on the surface as well as the fluid transport and mixing processes in the subsurface. Such subsurface-surface interactions would enable the transient development of various organizations of methane cycling in sulfate-deficient terrestrial mud volcanoes.

### Conflict of interest statement

The authors declare that the research was conducted in the absence of any commercial or financial relationships that could be construed as a potential conflict of interest.
