# Supplementary material for: Spatial variations of community structures and methane cycling across a transect of Lei-Gong-Hou mud volcanoes in eastern Taiwan
Source: Front Microbiol. 2014 Mar 25;5:121. doi: 10.3389/fmicb.2014.00121 (PMC3971192; doi:10.3389/fmicb.2014.00121)
Supplement: Supplementary file 1 [file DataSheet1.PDF]

Supplementary Table 1. Information for the bacterial sequences obtained in this study and Chang et al. (2012) (seqeucnes from bubbling fluids and core LGH02-c1).

OTU name	Phylum/Division	Order or below	BLAST closest sequence	Similarity	Accession No.	bubbling fluid	c1-3 cm	c1-7 cm	c1-9 cm	c1-15 cm	c1-21 cm	c1-29 cm	c2-0.5 cm	c2-2 cm	c2-4 cm	c2-6 cm	c2-15 cm	c3-2 cm	c3-11 cm	c3-19 cm	Sum
LGH02-B-024	Deltaproteobacteria	Desulfuromonadales	Pelobacter acetylenicus strain WoAcy1	98%	NR_029238.1	3	4	0	6	4	4	5	1	0	1	3	4	1	1	0	37
LGH02-B-052	Bacteroidetes	Unclassified	Bacteroidetes bacterium enrichment culture clone DT-1962	98%	AM292393.1	4	27	3	2	5	1	20	2	17	33	27	2	11	8	1	163
LGH02-B-002	Alphaproteobacteria	Rhodospirillales	Caenispirillum sp. HMD3183	99%	GU339179.1	1	0	1	1	0	2	1	0	1	2	1	1	0	2	0	13
LGH02-B-022	Deltaproteobacteria	Desulfuromonadales	Desulfuromonas michiganensis strain BB1	95%	AF357915.2	2	27	15	8	6	4	15	0	0	2	2	9	7	5	0	102
LGH02-B-055	Bacteroidetes	Unclassified	Uncultured bacterium clone PMMV-Bac265	95%	AJ937700.1	2	2	0	1	0	0	0	0	0	0	2	2	0	1	0	10
LGH02-B-142	Bacteroidetes	Unclassified	Uncultured Bacteriodetes bacterium clone CS-39-10 16S	95%	GQ406154.1	0	0	0	0	1	1	0	0	1	0	1	0	0	0	1	5
LGH02-B-090	Cyanobacteria	Unclassified	Uncultured cyanobacterium clone TDNP_Bbc97_2_47_54	97%	FJ516796.1	1	1	2	1	1	0	5	36	8	5	2	1	1	2	7	73
LGH02-B-053	Bacteroidetes	Order Incertae Sedis;Family Incertae Sedis;Prolixibacter;	Bacterium Phenol-4	99%	AF121885.1	1	7	6	4	3	3	3	0	5	8	3	4	8	12	1	68
LGH02-B-013	Gammaproteobacteria	Alteromonadales	Marinobacter sp. BR-13	99%	EU399549.1	8	1	1	7	9	16	3	7	0	0	1	6	0	2	1	62
LGH02-B-100	Lentisphaerae	Victivallales	Uncultured Verrucomicrobia Arctic95B-10	86%	AY028222.1	4	2	1	1	0	0	2	0	0	1	3	2	4	2	1	23
LGH02-B-011	Gammaproteobacteria	Alteromonadales	Marinobacter gudaonensis strain SL014B11A	99%	DQ629025.1	2	3	19	7	5	13	7	0	1	0	1	6	3	2	5	74
LGH02-B-103	Actinobacteria	Unclassified	Uncultured actinobacterium clone ANTXXIII_706-4_Bac6	98%	FN429805.1	1	0	0	0	0	0	0	0	0	0	0	1	0	0	1	3
LGH02-B-156	Gammaproteobacteria	Thiotrichales	Methylophaga thiooxidans strain DMS010	96%	DQ660915.1	0	0	0	0	1	1	0	0	0	0	0	1	0	0	0	3
LGH02-B-033	Deltaproteobacteria	Desulfuromonadales	Pelobacter seleniigenes strain KM	97%	DQ991964.1	0	0	0	0	0	2	1	0	0	0	0	1	0	0	0	4
LGH02-B-027	Deltaproteobacteria	Desulfobacterales	Uncultured bacterium clone KZNMV-25-B54	97%	FJ712587.1	1	1	0	0	1	0	0	0	0	0	0	11	0	0	0	14
LGH02-B-007	Alphaproteobacteria	Rhodospirillales	Uncultured alpha proteobacterium clone VS_CL-282	96%	FJ497534.1	0	0	0	1	1	0	0	0	1	0	0	1	0	0	0	4
LGH02-B-104	Actinobacteria	Unclassified	Uncultured actinobacterium clone D15_10	87%	EU266850.1	1	0	0	0	0	0	0	0	0	1	0	0	0	0	0	2
LGH02-B-025	Deltaproteobacteria	Desulfobacterales	Desulfopila aestuarii	97%	AB110542.2	2	2	0	0	1	1	0	0	1	0	0	5	0	1	0	13
LGH02-B-174	Firmicutes	Clostridiales	Uncultured bacterium clone SGUS1073	96%	FJ202130.1	0	0	0	0	2	5	0	13	9	3	0	1	0	0	0	33
LGH02-B-168	Deltaproteobacteria	Desulfobacterales	Desulfobacterium catecholicum strain NZva20	99%	NR_028895.1	0	0	0	0	1	1	0	0	0	0	1	0	1	0	0	4
LGH02-B-030	Deltaproteobacteria	Desulfuromonadales	Uncultured delta proteobacterium clone D50	97%	GQ249592.1	7	15	1	2	4	2	14	0	0	0	8	8	6	1	1	69
LGH02-B-150	Spirochaetes	Spirochaetales	Spirochaeta sp. SR	96%	FJ380060.1	0	0	0	0	0	0	0	1	7	1	2	0	0	1	0	12
LGH02-B-070	Firmicutes	Clostridiales	Uncultured Firmicutes bacterium clone NRB19	95%	HM041936.1	0	0	1	1	0	2	0	0	0	0	0	4	0	2	0	10
LGH02-B-075	Firmicutes	Clostridiales	Uncultured Caminicella sp. clone TCB136x	98%	DQ647129.1	1	0	0	0	0	1	0	0	0	0	0	1	0	1	0	4
LGH02-B-175	Firmicutes	Clostridiales	Uncultured marine bacterium clone s5_8_I_18	96%	FN396772.1	0	0	0	0	0	1	0	0	0	0	1	0	0	0	0	2
LGH02-B-088	Firmicutes	Clostridiales	Uncultured Firmicutes bacterium clone NRB19	94%	HM041936.1	1	0	0	0	0	0	0	0	0	0	0	1	0	0	0	2
LGH02-B-169	Bacteroidetes	Flavobacteriales	Gramella forsetii KT0803	95%	CU207366.1	0	0	0	0	1	0	0	0	0	1	0	0	0	0	0	2
LGH02-B-061	Bacteroidetes	Order Incertae Sedis;Family Incertae Sedis;Prolixibacter;	Bacterium Phenol-4	97%	AF121885.1	0	1	0	0	0	0	0	0	0	2	1	0	2	0	0	6
LGH02-B-012	Gammaproteobacteria	Order Incertae Sedis;Family Incertae	Thiohalophilus thiocyanatoxydans strain HRhD 2	98%	DQ469584.1	4	0	1	0	14	9	4	0	0	0	0	4	0	2	20	58
LGH02-B-097	Spirochaetes	Spirochaetales	Uncultured Spirochaetales bacterium clone MS4-18	91%	GQ354919.1	0	2	0	0	0	1	1	0	7	0	0	0	0	1	0	12
LGH02-B-098	Spirochaetes	Spirochaetales	Spirochaeta asiatica strain Z-7591	89%	NR_026300.1	0	0	0	0	1	0	1	0	3	0	0	0	0	0	0	5
LGH02-B-087	Firmicutes	Clostridiales	Uncultured bacterium clone SGUS604	96%	FJ202769.1	1	0	0	0	0	0	0	0	0	0	0	0	6	0	0	7
LGH02-B-145	Bacteroidetes	Cytophagales	Flexibacter tractuosus strain:IFO 15981	96%	AB078071.1	0	0	0	0	0	0	0	0	0	0	0	0	2	1	0	3
LGH02-B-056	Bacteroidetes	Unclassified	Uncultured Bacteroidetes bacterium clone 4-191	93%	GQ354965.1	0	0	0	1	0	0	0	0	0	0	0	0	2	1	0	4
LGH02-B-054	Bacteroidetes	Bacteroidales	Uncultured organism clone MAT-CR-P3-D08	98%	EU246102.1	0	1	3	3	0	0	2	1	0	0	0	0	4	2	0	16
LGH02-B-050	Epsilonproteobacteria	Campylobacterales	Arcobacter sp. CpA_b6	96%	FN397894.1	2	0	0	0	1	4	0	1	0	0	0	0	0	0	0	8
LGH02-B-014	Gammaproteobacteria	Order Incertae Sedis;Family Incertae	Thiohalophilus thiocyanatoxydans strain HRhD 2	96%	DQ469584.1	3	1	0	0	0	0	0	0	1	0	0	0	0	1	2	8
LGH02-B-137	Deltaproteobacteria	Desulfuromonadales	Desulfuromonas michiganensis strain BB1	94%	AF357915.2	0	0	0	0	0	0	0	0	0	0	0	0	1	0	1	2
LGH02-B-003	Alphaproteobacteria	Rhodobacterales	Roseobacter sp. LA9	96%	AF513439.2	1	1	0	0	2	2	0	0	0	0	0	0	1	0	1	8
LGH02-B-071	Firmicutes	Clostridiales	Uncultured bacterium clone DR546BH1103001SAD84 16S	95%	DQ234652.1	0	1	0	0	1	0	1	0	1	0	0	0	0	1	0	5
LGH02-B-129	Gammaproteobacteria	Vibrionales	Vibrio sp. QY102	99%	AY174868.1	0	0	0	0	0	0	0	0	1	0	0	0	0	0	1	2
LGH02-B-001	Alphaproteobacteria	Rhodospirillales	Thalassospira profundimaris strain WP0211	99%	AY186195.1	0	1	9	7	0	0	3	1	1	0	0	0	0	1	2	25
LGH02-B-069	Firmicutes	Clostridiales	Uncultured bacterium clone DR546BH1103001SAD84 16S	95%	DQ234652.1	1	0	0	2	0	5	0	0	1	0	0	0	0	1	0	10
LGH02-B-006	Alphaproteobacteria	Rhodobacterales	Rhodobacteraceae bacterium JAMH 0111	97%	AB524073.1	0	0	0	0	0	2	1	1	0	0	0	0	0	0	0	4
LGH02-B-057	Bacteroidetes	Unclassified	Uncultured organism clone MAT-CR-P2-A02	94%	EU246036.1	0	0	0	0	0	0	1	0	0	0	0	0	1	0	0	2
LGH02-B-173	Firmicutes	Clostridiales	Uncultured bacterium clone DR546BH1103001SAD84 16S	99%	DQ234652.1	0	0	0	0	0	3	0	0	0	0	0	0	1	0	0	4
LGH02-B-066	Firmicutes	Clostridiales	Uncultured Firmicutes bacterium clone GoM_GC232_4463	94%	AM745211.1	3	1	2	6	0	0	5	0	0	0	0	0	1	1	6	25
LGH02-B-177	Firmicutes	Clostridiales	Fusibacter paucivorans strain SEBR 4211	95%	NR_024886.1	0	0	0	0	0	0	0	0	5	1	1	0	0	0	0	7
LGH02-B-178	Unclassified	Unclassified	Uncultured bacterium clone 8M69	91%	JF272052	0	0	0	0	0	0	0	16	2	0	0	0	0	0	0	18
LGH02-B-179	Bacteroidetes	Flavobacteriales	Muricauda sp. 7IX/A01/155	99%	AY576744.1	0	0	0	0	0	0	0	0	2	0	0	1	0	0	0	3
LGH02-B-180	Alphaproteobacteria	Rhodospirillales	Caenispirillum sp. AK4	98%	FN995238.2	0	0	0	0	0	0	0	0	1	0	0	2	0	0	0	3
LGH02-B-181	Alphaproteobacteria	Rhodospirillales	Thalassospira sp. p67	91%	EU864264.1	0	0	0	0	0	0	0	0	1	1	0	0	0	0	0	2
LGH02-B-182	Betaproteobacteria	Burkholderiales	Limnobacter sp. DG1290	95%	DQ486503.1	0	0	0	0	0	0	0	0	1	0	0	0	1	0	0	2
LGH02-B-183	Bacteroidetes	Flavobacteriales	Muricauda sp. Bo10-09	98%	EU839357.1	0	0	0	0	0	0	0	0	1	2	0	0	0	0	0	3
LGH02-B-184	Planctomycetes	MSBL9	uncultured bacterium PMMV-Bac19	96%	AJ937688.1	0	0	0	0	0	0	0	0	0	0	0	2	0	0	0	2
LGH02-B-185	Firmicutes	Clostridiales	Clostridiales bacterium r28	96%	AB470960.1	0	0	0	0	0	0	0	0	0	0	0	2	0	0	0	2
LGH02-B-186	Gammaproteobacteria	Methylococcales	Methylomicrobium japanense strain NI	94%	NR_043450.1	0	0	0	0	0	0	0	0	0	0	0	1	0	0	0	1
LGH02-B-187	Alphaproteobacteria	Rhizobiales	Ochrobactrum sp. JCM 5503	99%	AB685698.1	0	0	0	0	0	0	0	0	0	0	0	1	0	0	0	1
LGH02-B-188	OP11	Unclassified	uncultured candidate division OP11 bacterium clone Bol26	89%	AY193135.1	0	0	0	0	0	0	0	0	0	0	0	1	0	0	0	1
LGH02-B-189	Gammaproteobacteria	Pseudomonadales	Acinetobacter baumannii strain IP026	98%	FJ009590.1	0	0	0	0	0	0	0	0	0	0	0	1	0	0	0	1
LGH02-B-190	Firmicutes	Clostridiales	Clostridia bacterium S710(0)-1	95%	GU136592.1	0	0	0	0	0	0	0	0	0	0	0	1	0	0	0	1
LGH02-B-191	Deltaproteobacteria	Desulfuromonadales	Uncultured delta proteobacterium clone D50	92%	GQ249592.1	0	0	0	0	0	0	0	0	0	0	4	0	0	0	0	4
LGH02-B-192	Alphaproteobacteria	Rhodospirillales	Marispirillum indicum strain B142	95%	NR_044545.1	0	0	0	0	0	0	0	0	0	0	2	0	0	0	0	2
LGH02-B-193	OP9	Unclassified	Uncultured bacterium clone B103E10	98%	FJ455886.1	0	0	0	0	0	0	0	0	0	0	2	0	0	0	0	2
LGH02-B-194	Deltaproteobacteria	Desulfuromonadales	Uncultured delta proteobacterium clone D50	97%	GQ249592.1	0	0	0	0	0	0	0	0	0	0	1	0	0	0	0	1

LGH02-B-195	Spirochaetes	Spirochaetales	Spirochaeta isovalerica strain DSM 2461T, clone 2	89%	FR749931.1	0	0	0	0	0	0	0	0	0	1	0	0	0	0	0	1
LGH02-B-196	Bacteroidetes	Unclassified	Uncultured bacterium clone PMMV-Bac265	94%	AJ937700.1	0	0	0	0	0	0	0	0	0	1	0	0	0	0	0	1
LGH02-B-197	Deltaproteobacteria	Bdellovibrionales	Bacteriovorax sp. NF3	99%	EF092443.2	0	0	0	0	0	0	0	0	0	1	0	0	0	0	0	1
LGH02-B-198	Chloroflexi	Anaerolineales	Uncultured Chloroflexi bacterium clone BC_CK775	94%	HQ727651.1	0	0	0	0	0	0	0	0	1	0	0	0	0	0	0	1
LGH02-B-199	Spirochaetes	Spirochaetales	Uncultured organism clone SBYC_3548	98%	JN443733.1	0	0	0	0	0	0	0	0	0	0	0	0	1	0	0	1
LGH02-B-200	Bacteroidetes	Unclassified	Uncultured organism clone SBZP_3824	99%	JN537161.1	0	0	0	0	0	0	0	0	0	0	0	0	1	0	0	1
LGH02-B-201	Bacteroidetes	Order Incertae Sedis;Family Incertae Sedis;Prolixibacter;	bacterium Phenol-4	98%	AF121885.1	0	0	0	0	0	0	0	0	0	0	0	0	1	0	0	1
LGH02-B-202	Firmicutes	Clostridiales	Dehalobacter sp. enrichment culture clone 06-1235251-121	89%	JQ599637.1	0	0	0	0	0	0	0	0	0	0	0	0	1	0	0	1
LGH02-B-203	Deltaproteobacteria	Desulfuromonadales	Delta proteobacterium EtOHpelo	94%	AY771935.1	0	0	0	0	0	0	0	0	0	0	0	0	1	0	0	1
LGH02-B-204	Bacteroidetes	Bacteroidales	Uncultured organism clone MAT-CR-P3-E07	96%	EU246108.1	0	0	0	0	0	0	0	0	0	0	0	0	1	0	0	1
LGH02-B-205	Gammaproteobacteria	Alteromonadales	Idiomarina sp. DF-B6	95%	EU515224.1	0	0	0	0	0	0	0	0	0	0	0	0	1	0	0	1
LGH02-B-206	Alphaproteobacteria.	Rhodospirillales	magnetite-containing magnetic vibrio	96%	L06455.1	0	0	0	0	0	0	0	0	0	0	0	0	1	0	0	1
LGH02-B-172	Firmicutes	Clostridiales	Clostridium viride strain DSM 6836	95%	NR_026204.1	0	0	0	0	0	2	0	0	0	0	0	0	0	0	0	2
LGH02-B-067	Firmicutes	Clostridiales	Clostridium sp. JC3 gene	91%	AB093546.1	8	1	0	1	0	0	5	0	0	0	0	0	0	0	0	15
LGH02-B-068	Firmicutes	Clostridiales	Uncultured marine bacterium clone s5_8_I_18	94%	FN396772.1	1	0	5	7	0	0	0	0	0	0	0	0	0	0	0	13
LGH02-B-049	Acidobacteria	Unclassified	Uncultured bacterium clone Acs2P38	95%	AB292962.1	1	3	0	3	1	2	0	0	0	0	0	0	0	0	0	10
LGH02-B-111	Nitrospirae	Nitrospirales	Uncultured Nitrospirae bacterium clone MSB-4D12	91%	DQ811893.1	4	0	0	1	0	0	0	0	0	0	0	0	0	0	0	5
LGH02-B-051	Zetaproteobacteria	Mariprofundales	Mariprofundus ferrooxydans strain JV-1	94%	EF493244.1	1	0	1	0	0	1	0	0	0	0	0	0	0	0	0	3
LGH02-B-102	Actinobacteria	Micrococcales	Cellulomonas sp. NP4	99%	EU196315.1	0	2	0	0	0	0	0	0	0	0	0	0	0	1	0	3
LGH02-B-125	Gammaproteobacteria	Thiotrichales	Methylophaga thiooxidans strain DMS010	96%	DQ660915.1	0	0	0	0	0	0	0	0	0	0	0	0	0	0	4	4
LGH02-B-136	Deltaproteobacteria	Desulfobacterales	Desulfurivibrio alkaliphilus strain AHT2	91%	EF422413.1	0	0	0	0	0	1	0	0	0	0	0	0	0	0	7	8
LGH02-B-120	Alphaproteobacteria.	Rhizobiales	Uncultured alpha proteobacterium clone SV-4	95%	GU233828.1	0	0	0	0	0	0	0	0	0	0	0	0	0	6	0	6
LGH02-B-026	Deltaproteobacteria	Desulfobacterales	Delta proteobacterium PL12	98%	AB468588.1	0	0	0	1	1	1	0	0	0	0	0	0	0	1	1	5
LGH02-B-126	Gammaproteobacteria	Methylococcales	Methylosarcina lacus strain LW14	93%	AY007296.1	0	0	0	0	0	0	0	0	0	0	0	0	0	2	2	4
LGH02-B-121	Alphaproteobacteria.	Rhodobacterales	Rhodobacteraceae bacterium DG1297	96%	DQ486508.1	0	0	0	0	0	2	0	0	0	0	0	0	0	0	2	4
LGH02-B-151	Deferribacteres	Deferribacterales	Deferribacter autotrophicus strain SL50	87%	EU407777.1	0	0	0	0	0	0	0	0	0	0	0	0	0	2	0	2
LGH02-B-091	Chloroflexi	Caldilineales	Uncultured Chloroflexi bacterium clone HAHS13.6	98%	HQ397021.1	2	1	1	0	0	0	0	0	0	0	0	0	0	0	0	4
LGH02-B-127	Gammaproteobacteria	Chromatiales	Alkalilimnicola ehrlichii MLHE-1	94%	AF406554.1	0	0	0	0	0	0	0	0	0	0	0	0	0	0	3	3
LGH02-B-128	Gammaproteobacteria	Unclassified	Uncultured Chromatiales bacterium clone TDNP_Wbc97_2	95%	FJ517068.1	0	0	0	0	0	1	0	0	0	0	0	0	0	0	3	4
LGH02-B-092	Chloroflexi	Unclassified	Uncultured bacterium clone SK27B-40	99%	AB300063.1	2	0	0	0	0	0	0	0	0	0	0	0	0	0	0	2
LGH02-B-072	Firmicutes	Clostridiales	Uncultured bacterium clone DR546BH1103001SAD84	92%	DQ234652.1	0	1	0	0	0	0	0	0	0	0	0	0	0	1	0	2
LGH02-B-004	Alphaproteobacteria.	Rhodospirillales	Uncultured alpha proteobacterium clone SGTA560	94%	GQ348409.1	2	0	0	0	0	0	0	0	0	0	0	0	0	0	0	2
LGH02-B-073	Firmicutes	Clostridiales	Uncultured Firmicutes bacterium clone GoM_GC232_4463	96%	AM745211.1	0	0	0	1	0	0	1	0	0	0	0	0	0	0	0	2
LGH02-B-074	Firmicutes	Clostridiales	Uncultured Firmicutes bacterium clone eub623C9	94%	GQ390236.1	1	0	0	1	0	0	0	0	0	0	0	0	0	0	0	2
LGH02-B-029	Deltaproteobacteria	Desulfobacterales	Desulfobacterium catecholicum strain NZva20	99%	NR_028895.1	0	0	1	0	0	0	1	0	0	0	0	0	0	0	0	2
LGH02-B-164	Deltaproteobacteria	Desulfuromonadales	Desulfuromonas michiganensis strain BB1	94%	AF357915.2	0	0	0	0	0	1	0	0	0	0	0	0	0	0	0	1
LGH02-B-158	Gammaproteobacteria	Oceanospirillales	Saccharospirillum salsuginis strain YIM-Y25	99%	EF177670.1	0	0	0	0	0	1	0	0	0	0	0	0	0	0	0	1
LGH02-B-159	Gammaproteobacteria	Alteromonadales	Marinimicrobium haloxylanilyticum strain SX15	95%	GQ920839.1	0	0	0	0	0	1	0	0	0	0	0	0	0	0	0	1
LGH02-B-165	Deltaproteobacteria	Syntrophobacterales	Uncultured bacterium clone GoC_Bac_87_D0_C1_M0	93%	FJ813565.1	0	0	0	0	0	1	0	0	0	0	0	0	0	0	0	1
LGH02-B-166	Deltaproteobacteria	Desulfarculales	Uncultured bacterium clone MD2896-B265	94%	EU385822.1	0	0	0	0	0	1	0	0	0	0	0	0	0	0	0	1
LGH02-B-157	Gammaproteobacteria	Chromatiales	Halothiobacillus hydrothermalis strain R3	99%	NR_025943.1	0	0	0	0	0	2	0	0	0	0	0	0	0	0	0	2
LGH02-B-176	Firmicutes	Clostridiales	Alkalibacter saccharofermentans strain Z-79820	98%	AY312403.2	0	0	0	0	0	1	0	0	0	0	0	0	0	0	0	1
LGH02-B-167	Deltaproteobacteria	Desulfobacterales	Uncultured bacterium clone BP93	98%	HQ190554.1	0	0	0	0	0	1	0	0	0	0	0	0	0	0	0	1
LGH02-B-153	Alphaproteobacteria.	Rhodobacterales	Maritimibacter alkaliphilus strain HTCC2654	97%	DQ915443.3	0	0	0	0	0	1	0	0	0	0	0	0	0	0	0	1
LGH02-B-160	Gammaproteobacteria	Aeromonadales	Oceanimonas smirnovii strain SS2	98%	GU980766.1	0	0	0	0	0	1	0	0	0	0	0	0	0	0	0	1
LGH02-B-161	Gammaproteobacteria	Order Incertae Sedis;Family Incertae Sedis;Marinicella;	Uncultured Chromatiales bacterium clone TDNP_Wbc97_2	98%	FJ517068.1	0	0	0	0	0	1	0	0	0	0	0	0	0	0	0	1
LGH02-B-162	Gammaproteobacteria	Unclassified	Uncultured bacterium clone HSS157	98%	HQ397439.1	0	0	0	0	1	0	0	0	0	0	0	0	0	0	0	1
LGH02-B-163	Gammaproteobacteria	Alteromonadales	Idiomarina baltica strain SS-01	96%	EU624441.1	0	0	0	0	1	0	0	0	0	0	0	0	0	0	0	1
LGH02-B-154	Alphaproteobacteria.	Rhodospirillales	Thalassobaculum litoreum strain CL-GR58	99%	EF203900.2	0	0	0	0	1	0	0	0	0	0	0	0	0	0	0	1
LGH02-B-170	Bacteroidetes	Order III Incertae Sedis;ML602J-37;	Uncultured Bacteroidetes bacterium clone Y268	96%	EU328106.1	0	0	0	0	1	0	0	0	0	0	0	0	0	0	0	1
LGH02-B-155	Alphaproteobacteria.	Rhodospirillales	Thalassospira profundimaris strain WP0211	94%	AY186195.1	0	0	0	0	2	3	0	0	0	0	0	0	0	0	0	5
LGH02-B-171	Bacteroidetes	Order III Incertae Sedis;Family Incertae	Sphingobacteriales bacterium KIP gene	98%	AB540002.1	0	0	0	0	1	0	0	0	0	0	0	0	0	0	0	1
LGH02-B-031	Deltaproteobacteria	Unclassified	Uncultured bacterium clone KZNMV-25-B54	92%	FJ712587.1	0	0	0	0	0	0	1	0	0	0	0	0	0	0	0	1
LGH02-B-005	Alphaproteobacteria.	Rhodospirillales	Caenispirillum sp. HMD3183	98%	GU339179.1	0	0	0	0	0	0	1	0	0	0	0	0	0	0	0	1
LGH02-B-093	Chloroflexi	Anaerolineales	Uncultured Chloroflexi bacterium clone I1G	93%	FJ205347.1	0	0	0	0	0	0	1	0	0	0	0	0	0	0	0	1
LGH02-B-077	Firmicutes	Clostridiales	Uncultured Firmicutes bacterium clone GoM_GC232_4463	96%	AM745211.1	0	0	0	0	0	0	1	0	0	0	0	0	0	0	0	1
LGH02-B-117	OP11	Unclassified	Uncultured candidate division OP11clone BMS61	96%	AY193167.1	0	0	0	0	0	0	1	0	0	0	0	0	0	0	0	1
LGH02-B-078	Firmicutes	Clostridiales	Uncultured Firmicutes bacterium clone NRB19	95%	HM041936.1	0	0	0	0	0	0	1	0	0	0	0	0	0	0	0	1
LGH02-B-032	Deltaproteobacteria	Desulfuromonadales	Pelobacter acetylenicus strain WoAcy1	98%	NR_029238.1	0	0	0	0	0	0	1	0	0	0	0	0	0	0	0	1
LGH02-B-058	Bacteroidetes	Unclassified	Uncultured organism clone MAT-CR-P3-D08	93%	EU246102.1	0	0	0	0	0	0	1	0	0	0	0	0	0	0	0	1
LGH02-B-059	Bacteroidetes	Unclassified	Bacteroidetes bacterium enrichment culture clone DT-1962	97%	AM292393.1	0	0	0	0	0	0	1	0	0	0	0	0	0	0	0	1
LGH02-B-016	Gammaproteobacteria	Alteromonadales	Marinobacter sp. BR-13	99%	EU399549.1	0	0	0	1	0	0	0	0	0	0	0	0	0	0	0	1
LGH02-B-034	Deltaproteobacteria	Unclassified	Uncultured bacterium clone WC30	93%	GQ461684	0	0	0	1	0	0	0	0	0	0	0	0	0	0	0	1
LGH02-B-079	Firmicutes	Clostridiales	Uncultured marine bacterium clone s5_8_I_18	97%	FN396772.1	0	0	0	1	0	0	0	0	0	0	0	0	0	0	0	1
LGH02-B-035	Deltaproteobacteria	Desulfuromonadales	Pelobacter acetylenicus strain WoAcy1	98%	NR_029238.1	0	0	0	1	0	0	0	0	0	0	0	0	0	0	0	1
LGH02-B-008	Alphaproteobacteria	Rhodospirillales	Thalassospira profundimaris strain WP0211	99%	AY186195.1	0	0	0	1	0	0	0	0	0	0	0	0	0	0	0	1
LGH02-B-017	Gammaproteobacteria	Alteromonadales	Idiomarina loihiensis strain L2-TR	99%	NR_025119.1	0	0	0	1	0	2	0	0	0	0	0	0	0	0	0	3
LGH02-B-080	Firmicutes	Clostridiales	Uncultured marine bacterium clone s5_8_I_18	96%	FN396772.1	0	0	0	1	0	0	0	0	0	0	0	0	0	0	0	1
LGH02-B-037	Deltaproteobacteria	Desulfuromonadales	Desulfuromonas michiganensis strain BB1	96%	AF357915.2	0	0	0	1	0	0	0	0	0	0	0	0	0	0	0	1

LGH02-B-038	Deltaproteobacteria	Desulfovibrionales	Desulfovibrio aespoeensis clone Aspo3	98%	EU680957.1	0	0	0	1	0	0	0	0	0	0	0	0	0	0	0	1
LGH02-B-039	Deltaproteobacteria	Desulfuromonadales	Uncultured Desulfuromonas sp. clone B93	95%	EU328027.1	0	0	0	1	0	0	0	0	0	0	0	0	0	0	0	1
LGH02-B-081	Firmicutes	Clostridiales	Uncultured marine bacterium clone s5_8_I_18	92%	FN396772.1	0	0	1	0	0	0	0	0	0	0	0	0	0	0	0	1
LGH02-B-082	Firmicutes	Clostridiales	Dehalobacterium formicoaceticum	94%	X86690.1	0	0	1	0	0	0	0	0	0	0	0	0	0	0	0	1
LGH02-B-060	Bacteroidetes	Unclassified	Uncultured organism clone MAT-CR-P3-C07	93%	EU246095.1	0	0	1	0	0	0	0	0	0	0	0	0	0	0	0	1
LGH02-B-018	Gammaproteobacteria	Alteromonadales	Marinobacter gudaonensis strain SL014B11A	99%	NR_025119.1	0	0	2	0	0	0	0	0	0	0	0	0	0	0	0	2
LGH02-B-083	Firmicutes	Clostridiales	Uncultured Clostridium sp. clone U50	99%	GU255485.1	0	0	1	0	0	0	0	0	0	0	0	0	0	0	0	1
LGH02-B-020	Gammaproteobacteria	Alteromonadales	Marinobacter gudaonensis strain SL014B11A	99%	DQ629025.1	0	0	1	0	0	0	0	0	0	0	0	0	0	0	0	1
LGH02-B-085	Firmicutes	Unclassified	Moorella perchloratireducens 16S	90%	EF060194.1	0	0	1	0	0	0	0	0	0	0	0	0	0	0	0	1
LGH02-B-040	Deltaproteobacteria	Desulfuromonadales	Uncultured delta proteobacterium clone D50	97%	GQ249592.1	0	1	0	0	0	0	0	0	0	0	0	0	0	0	0	1
LGH02-B-062	Bacteroidetes	Unclassified	Uncultured bacterium clone SINH522	95%	HM128071.1	0	1	0	0	0	0	0	0	0	0	0	0	0	0	0	1
LGH02-B-094	Chloroflexi	Anaerolineales	Uncultured Chloroflexi bacterium clone MSB-5B1	94%	DQ811877.1	0	1	0	0	0	0	0	0	0	0	0	0	0	0	0	1
LGH02-B-009	Alphaproteobacteria	Rhodospirillales	Caenispirillum sp. HMD3183	99%	GU339179.1	0	1	0	0	0	0	0	0	0	0	0	0	0	0	0	1
LGH02-B-086	Firmicutes	Clostridiales	Uncultured Firmicutes bacterium clone GoM_GC232_4463	96%	AM745211.1	0	1	0	0	0	0	0	0	0	0	0	0	0	0	0	1
LGH02-B-041	Deltaproteobacteria	Desulfuromonadales	Uncultured delta proteobacterium clone D50	96%	GQ249592.1	0	1	0	0	0	0	0	0	0	0	0	0	0	0	0	1
LGH02-B-010	Alphaproteobacteria	Rhodospirillales	Marispirillum indicum strain B142	95%	EU642410.1	0	1	0	0	0	0	0	0	0	0	0	0	0	0	0	1
LGH02-B-042	Deltaproteobacteria	Unclassified	Uncultured delta proteobacterium clone 70 EDB2	91%	AM882621.1	0	1	0	0	0	0	0	0	0	0	0	0	0	0	0	1
LGH02-B-101	Lentisphaerae	Unclassified	Uncultured Verrucomicrobia Arctic95B-10	87%	AY028222.1	0	1	0	0	0	0	0	0	0	0	0	0	0	0	0	1
LGH02-B-063	Bacteroidetes	Order III Incertae Sedis;ML602J-37;	Bacteroidetes bacterium MO54 16S	96%	AY553122.1	0	1	0	0	1	0	0	0	0	0	0	0	0	0	0	2
LGH02-B-064	Bacteroidetes	Cytophagales	Uncultured Flammovirgaceae bacterium clone TDNP_Wbc	95%	FJ517065.1	0	1	0	0	0	0	0	0	0	0	0	0	0	0	0	1
LGH02-B-138	Deltaproteobacteria	Desulfobacterales	Uncultured Desulfocapsa sp. clone SB1_88	95%	AY177798.1	0	0	0	0	0	0	0	0	0	0	0	0	0	0	1	1
LGH02-B-130	Gammaproteobacteria	Oceanospirillales	Uncultured bacterium clone SINP601	96%	HM127577.1	0	0	0	0	0	0	0	0	0	0	0	0	0	0	1	1
LGH02-B-139	Deltaproteobacteria	Desulfobacterales	Desulfotignum toluenicum strain H3 clone 7	97%	EF207159.1	0	0	0	0	0	0	0	0	0	0	0	0	0	0	1	1
LGH02-B-122	Alphaproteobacteria	Rickettsiales	Uncultured alpha proteobacterium clone ADK-BTh02-10	94%	EF520397.1	0	0	0	0	0	0	0	0	0	0	0	0	0	0	1	1
LGH02-B-131	Gammaproteobacteria	Order Incertae Sedis;Family Incertae	Uncultured bacterium clone FeSO4_B_166	95%	GQ356981.1	0	0	0	0	0	0	0	0	0	0	0	0	0	0	1	1
LGH02-B-132	Gammaproteobacteria	Methylococcales	Methylobacter sp. LW1	92%	AF150784.1	0	0	0	0	0	0	0	0	0	0	0	0	0	0	1	1
LGH02-B-133	Gammaproteobacteria	Order Incertae Sedis;Family Incertae	Uncultured bacterium clone FeSO4_B_166	95%	GQ356981.1	0	0	0	0	0	0	0	0	0	0	0	0	0	0	1	1
LGH02-B-143	Bacteroidetes	Unclassified	Uncultured Bacteroidetes bacterium clone MSB-3B7 16S	94%	DQ811907.1	0	0	0	0	0	0	0	0	0	0	0	0	0	0	1	1
LGH02-B-144	Bacteroidetes	Cytophagales	Sphingobacteria bacterium JAM-BA0302	93%	AB362263.1	0	0	0	0	0	0	0	0	0	0	0	0	0	1	0	1
LGH02-B-123	Alphaproteobacteria	Rhodospirillales	Uncultured bacterium gene for 16S clone: YWB01	96%	AB294270.1	0	0	0	0	0	0	0	0	0	0	0	0	0	1	0	1
LGH02-B-141	Unclassified	Unclassified	Delta proteobacterium EtOHpelo	85%	AY771935.1	0	0	0	0	0	0	0	0	0	0	0	0	0	1	0	1
LGH02-B-152	OP9	Unclassified	Uncultured candidate division JS1 bacterium clone Hyd01-1	97%	AJ535219.1	0	0	0	0	0	0	0	0	0	0	0	0	0	1	0	1
LGH02-B-149	Spirochaetes	Spirochaetales	Uncultured spirochete clone LF006 16S	92%	AY605159.1	0	0	0	0	0	0	0	0	0	0	0	0	0	1	0	1
LGH02-B-146	Firmicutes	Clostridiales	Clostridia bacterium S710(0)-1	94%	GU136592.1	0	0	0	0	0	0	0	0	0	0	0	0	0	1	0	1
LGH02-B-134	Gammaproteobacteria	Alteromonadales	Marinobacter gudaonensis strain SL014B11A	99%	DQ629025.1	0	0	0	0	0	0	0	0	0	0	0	0	0	1	0	1
LGH02-B-135	Gammaproteobacteria	Vibrionales	Vibrio sp. S1110	99%	FJ457366.1	0	0	0	0	1	0	0	0	0	0	0	0	0	1	0	2
LGH02-B-147	Firmicutes	Clostridiales	Clostridiales bacterium r28	95%	AB470960.1	0	0	0	0	0	0	0	0	0	0	0	0	0	1	0	1
LGH02-B-140	Deltaproteobacteria	Desulfobacterales	Desulfobacterium corrodens	95%	AY274450.1	0	0	0	0	0	0	0	0	0	0	0	0	0	1	0	1
LGH02-B-124	Alphaproteobacteria	Unclassified	Uncultured bacterium clone BD72BR112	96%	GU362941.1	0	0	0	0	0	0	0	0	0	0	0	0	0	1	0	1
LGH02-B-112	Nitrospirae	Nitrospirales	Uncultured Nitrospirae bacterium clone MSB-4D12	91%	DQ811893.1	1	0	0	0	0	0	0	0	0	0	0	0	0	0	0	1
LGH02-B-043	Deltaproteobacteria	Desulfarculales	Uncultured bacterium clone MD2896-B265	96%	EU385822.1	1	0	0	0	0	0	0	0	0	0	0	0	0	0	0	1
LGH02-B-021	Gammaproteobacteria	Unclassified	Methylococcus capsulatus str. Bath	93%	AE017282.2	1	0	0	0	0	0	0	0	0	0	0	0	0	0	0	1
LGH02-B-115	Planctomycetes	Unclassified	Uncultured bacterium clone MD2896-B125	94%	EU385728.1	1	0	0	0	0	0	0	0	0	0	0	0	0	0	0	1
LGH02-B-095	Chloroflexi	vadinBA26	Uncultured Dehalococcoides sp. clone JJB222	96%	GQ143775.1	1	0	0	0	0	0	0	0	0	0	0	0	0	0	0	1
LGH02-B-045	Deltaproteobacteria	Desulfobacterales	Uncultured delta proteobacterium clone Z195MB64 16S	90%	FJ484777.1	1	0	0	0	0	0	0	0	0	0	0	0	0	0	0	1
LGH02-B-116	Nitrospirae	Unclassified	Uncultured candidate division OP8 bacterium clone JJB113	96%	GQ143760.1	1	0	0	0	0	0	0	0	0	0	0	0	0	0	0	1
LGH02-B-065	Bacteroidetes	Cytophagales	Sphingobacteria bacterium JAM-BA0302	93%	AB362263.1	1	0	0	0	0	0	0	0	0	0	0	0	0	0	0	1
LGH02-B-113	Nitrospirae	Nitrospirales	Uncultured Nitrospirae bacterium clone MSB-4D12	91%	DQ811893.1	1	0	0	0	0	0	0	0	0	0	0	0	0	0	0	1
LGH02-B-096	Bacteria.BH180-139	Unclassified	Uncultured Chloroflexi bacterium gene clone: IODP1324B1	96%	AB448920.1	1	0	0	0	0	0	0	0	0	0	0	0	0	0	0	1
LGH02-B-099	Spirochaetes	Spirochaetales	Uncultured Spirochaetes bacterium clone QEDS3DD08	92%	CU921590.1	1	0	0	0	0	0	0	0	0	0	0	0	0	0	0	1
LGH02-B-105	Actinobacteria	Unclassified	Uncultured actinobacterium clone D15_10	89%	EU266850.1	1	0	0	0	0	0	0	0	0	0	0	0	0	0	0	1
LGH02-B-046	Deltaproteobacteria	DTB120	Uncultured delta proteobacterium clone 89	91%	FJ205262.1	1	0	0	0	0	0	0	0	0	0	0	0	0	0	0	1
LGH02-B-106	Actinobacteria	Unclassified	Uncultured actinobacterium clone 4-42	98%	AF351232.1	1	0	0	0	0	0	0	0	0	0	0	0	0	0	0	1
LGH02-B-089	Firmicutes	Clostridiales	Uncultured bacterium clone BS131	93%	AB240269.1	1	0	0	0	0	0	0	0	0	0	0	0	0	0	0	1
LGH02-B-107	Actinobacteria	Unclassified	Uncultured actinobacterium clone D15_10	87%	EU266850.1	1	0	0	0	0	0	0	0	0	0	0	0	0	0	0	1
LGH02-B-108	Gammaproteobacteria	BD2-11 terrestrial group	Uncultured bacterium clone MidBa36	97%	FJ748806.1	1	0	0	0	0	0	0	0	0	0	0	0	0	0	0	1
LGH02-B-110	Acidobacteria	Subgroup 3	Uncultured Acidobacterium sp. clone Z17M94B	94%	FJ484496.1	1	0	0	0	0	0	0	0	0	0	0	0	0	0	0	1
LGH02-B-114	Planctomycetes	MSBL9	Uncultured bacterium clone MD2902-B78	95%	EU385893.1	1	0	0	0	0	0	0	0	0	0	0	0	0	0	0	1
LGH02-B-047	Deltaproteobacteria	Unclassified	Uncultured delta proteobacterium clone OPPB013	86%	AY861756.1	1	0	0	0	0	0	0	0	0	0	0	0	0	0	0	1
LGH02-B-048	Deltaproteobacteria	DTB120	Uncultured delta proteobacterium clone 89	91%	FJ205262.1	1	0	0	0	0	0	0	0	0	0	0	0	0	0	0	1
LGH02-B-044	Deltaproteobacteria	Desulfarculales	Uncultured delta proteobacterium clone ccslm247 16S	95%	AY133092.1	1	0	0	0	0	0	0	0	0	0	0	0	0	0	0	1
Sum						101	120	81	86	76	113	111	80	80	67	69	88	72	79	82	1305
