# Supplementary material for: Spatial variations of community structures and methane cycling across a transect of Lei-Gong-Hou mud volcanoes in eastern Taiwan
Source: Front Microbiol. 2014 Mar 25;5:121. doi: 10.3389/fmicb.2014.00121 (PMC3971192; doi:10.3389/fmicb.2014.00121)
Supplement: Supplementary file 2 [file DataSheet2.PDF]

Supplementary Table 2. Informaiton for the archaeal sequences obtained in this study and Chang et al. (2012) (sequences from bubbling fluids and core LGH02-c1).

OTU name	Group	BLAST closest sequence	Similarity	Accession No.	bubbling fluid	c1-3 cm	c1-7 cm	c1-9 cm	c1-15 cm	c1-21 cm	c1-29 cm	c2-0.5 cm	c2-2 cm	c2-4 cm	c2-6 cm	c2-15 cm	c3-2 cm	c3-11 cm	c3-19 cm	Sum
LGH02-A-11	AAA	Uncultured archaeon clone SSA0101-1	94%	HM159357.1	5	2	0	1	2	1	2	3	1	2	2	1	0	0	13	35
LGH02-A-13	AAA	Uncultured archaeon clone MH1492_B10B	93%	EU155957.1	0	0	1	0	0	0	0	0	0	0	0	0	3	0	0	4
LGH02-A-15	AAA	Uncultured archaeon clone SSA0101-9	97%	EF644786.1	1	0	0	0	1	1	0	3	0	0	0	0	0	0	0	6
LGH02-A-14	AAA	Uncultured archaeon clone MH1492_B10B	96%	HM159365.1	1	0	0	0	0	1	0	0	0	0	0	0	0	0	1	3
LGH02-A-69	AAA	Uncultured sediment archaeon 16S rRNA gene, clone A285-	99%	FN553696.1	0	0	0	0	0	0	0	0	0	0	0	0	0	0	1	1
LGH02-A-12	AAA	Uncultured archaeon clone SSA0101-2	95%	HM159358.1	0	1	0	0	0	0	1	0	0	0	0	0	0	0	0	2
LGH02-A-33	ANME-1	Uncultured archaeon clone AHH11DNA_16	99%	AB554239.1	0	0	0	0	2	4	1	2	1	0	0	2	0	0	1	13
LGH02-A-34	ANME-1	Uncultured archaeon clone KZNMV-25-A3	97%	FJ712384.1	1	0	0	0	0	0	1	0	0	0	0	0	0	0	0	2
LGH02-A-25	ANME-2a	Archaeon enrichment culture clone AOM-Clone-D10	96%	FJ555678.1	27	11	3	4	5	3	13	5	9	5	5	2	1	1	9	103
LGH02-A-24	ANME-2a	Uncultured archaeon clone: ANME2aPC	97%	AB461390.1	32	34	25	6	24	11	21	9	13	6	23	8	2	3	19	236
LGH02-A-26	ANME-2a	Archaeon enrichment culture clone AOM-Clone-D10	97%	FJ555678.1	9	7	5	1	0	7	2	4	7	1	1	0	2	0	11	57
LGH02-A-27	ANME-2a	Archaeon enrichment culture clone AOM-Clone-D10	97%	FJ555678.1	1	0	0	0	0	0	1	2	0	0	0	0	0	0	1	5
LGH02-A-28	ANME-2a	Uncultured archaeon clone:OT-A17.11	97%	AB252424.1	0	0	0	0	1	0	1	0	1	0	0	0	0	0	0	3
LGH02-A-43	ANME-2a	Uncultured archaeon clone: ANME2aPC	93%	AB461390.1	0	0	0	0	1	0	0	0	0	0	0	0	0	0	0	1
LGH02-A-54	ANME-2a	archaeon enrichment culture clone AOM-Clone-D10	96%	FJ555678.1	0	0	0	0	0	0	0	0	0	0	0	1	0	0	0	1
LGH02-A-56	ANME-2a	uncultured archaeon clone: ANME2aPC	96%	AB461390.1	0	0	0	0	0	0	0	0	0	0	1	0	0	0	0	1
LGH02-A-66	ANME-2a	Uncultured archaeon clone: ANME2aPC	95%	AB461390.1	0	0	0	0	0	0	0	1	0	0	0	0	0	0	0	1
LGH02-A-42	ANME-2a	Uncultured archaeon clone Hyd24-Arch17	97%	AJ578113.1	0	0	0	0	0	1	0	0	0	0	0	0	0	0	0	1
LGH02-A-29	ANME-2a	Archaeon enrichment culture clone AOM-Clone-D10	96%	FJ555678.1	0	0	0	0	0	0	1	0	0	0	0	0	0	0	0	1
LGH02-A-30	ANME-2a	Archaeon enrichment culture clone AOM-Clone-D10	94%	FJ555678.1	0	0	1	0	0	0	0	0	0	0	0	0	0	0	0	1
LGH02-A-31	ANME-2a	Archaeon enrichment culture clone AOM-Clone-D10	93%	FJ555678.1	0	1	0	0	0	0	0	0	0	0	0	0	0	0	0	1
LGH02-A-65	DSEG	Uncultured archaeon clone Edge0arc2	88%	GU190985.1	0	0	0	0	0	0	0	1	0	0	0	0	0	0	0	1
LGH02-A-41	MBGB	Uncultured archaeon clone AMSMV-S1-A61	97%	FJ649533.1	0	0	0	0	0	1	0	0	0	0	0	0	0	0	2	3
LGH02-A-55	MBGB	Uncultured crenarchaeote clone GNA03H07	95%	EU732003.1	0	0	0	0	0	0	0	0	0	0	0	1	0	0	0	1
LGH02-A-57	MBGB	Uncultured archaeon clone Kazan-2A-04	94%	AY591982.1	0	0	0	0	0	0	0	0	0	0	1	0	0	0	0	1
LGH02-A-47	MCG	Uncultured archaeon clone HDBA_SISX542	95%	HM187502.1	0	0	0	0	1	0	0	0	0	1	0	0	0	0	0	2
LGH02-A-58	MCG	Uncultured archaeon GZK8	97%	AJ576209.1	0	0	0	0	0	0	0	0	0	1	0	0	0	0	0	1
LGH02-A-62	MCG	Uncultured archaeon clone 1H3M_ARC110	95%	JN229609.1	0	0	0	0	0	0	0	0	1	0	0	0	0	0	0	1
LGH02-A-64	MCG	Uncultured archaeon clone SAT_3G5	97%	FJ655692.1	0	0	0	0	0	0	0	1	0	0	0	0	0	0	0	1
LGH02-A-67	MCG	Uncultured archaeon clone 54BarcR55	96%	JN605044.1	0	0	0	0	0	0	0	1	0	0	0	0	0	0	0	1
LGH02-A-68	MCG	Uncultured archaeon clone 1H3CARC137	97%	JN229482.1	0	0	0	0	0	0	0	1	0	0	0	0	0	0	0	1
LGH02-A-35	MCG	Aarhus Bay (Henry IV/04) clone Arch43	98%	FN428813.1	0	1	1	0	1	0	1	0	0	0	0	0	0	0	0	4
LGH02-A-45	MCG	Uncultured archaeon clone SAT_3G5	97%	FJ655692.1	0	0	0	0	1	0	0	0	0	0	0	0	0	0	0	1
LGH02-A-46	MCG	Uncultured archaeon clone GZK8	96%	AJ576209.1	0	0	0	0	1	0	0	0	0	0	0	0	0	0	0	1
LGH02-A-38	MCG	Uncultured archaeon clone ZES-104	96%	EF367541.1	0	0	0	1	0	0	0	0	0	0	0	0	0	0	0	1
LGH02-A-39	MCG	Uncultured crenarchaeote clone LPBB59	95%	FJ902695.1	1	0	0	0	0	1	0	0	0	0	0	0	0	0	0	2
LGH02-A-17	Methanomicrobiales	Methanocalculus strain MHT-1	99%	NR_028148.1	1	3	2	7	8	4	7	4	9	13	11	8	2	6	1	86
LGH02-A-20	Methanomicrobiales	Methanoplanus petrolearius SEBR 4847	98%	NR_028240.1	0	0	0	0	0	1	1	1	7	2	0	1	0	8	1	22
LGH02-A-48	Methanomicrobiales	Methanoplanus petrolearius DSM 11571	93%	CP002117.1	0	0	0	0	0	0	0	0	0	0	0	0	3	1	0	4
LGH02-A-59	Methanomicrobiales	Methanocalculus pumilus	95%	AB008853.1	0	0	0	0	0	0	0	0	0	2	0	0	0	0	0	2
LGH02-A-23	Methanomicrobiales	Uncultured archaeon clone MidArch4	99%	EF680216.1	0	1	0	0	0	0	0	0	0	0	0	0	0	0	0	1
LGH02-A-01	Methanosarcinales	Methanococcoides methylutens DSM2657T	99%	FR733669.1	1	7	17	26	16	19	13	12	9	17	0	17	0	28	1	183
LGH02-A-40	Methanosarcinales	Methanosarcina semesiae MD1	97%	NR_028182.1	0	0	0	0	2	7	0	12	4	0	3	12	9	28	0	77
LGH02-A-05	Methanosarcinales	Uncultured archaeon clone KM07-Ea-3	99%	EU420698.1	2	0	5	5	2	4	7	2	1	0	1	0	12	16	0	57
LGH02-A-02	Methanosarcinales	Methanococcoides sp. strain NaT1	97%	Y16946.1	0	0	0	1	0	0	0	0	1	0	7	0	8	0	0	17
LGH02-A-10	Methanosarcinales	Methanosaeta sp. clone A1	98%	AJ133791.1	3	0	0	1	2	0	2	0	1	0	0	0	0	1	0	10
LGH02-A-44	Methanosarcinales	Uncultured archaeon clone Kazan-2A-32/BC19-2A-32	96%	AY592008.1	0	0	0	0	1	0	0	1	0	0	0	0	0	0	0	2
LGH02-A-06	Methanosarcinales	Methanosarcina semesiae MD1	96%	NR_028182.1	0	0	2	1	0	0	0	1	0	0	0	0	0	1	0	5
LGH02-A-22	Methanosarcinales	Uncultured archaeon clone KZNMV-25-A18	97%	FJ712387.1	2	0	0	0	1	1	1	0	0	0	0	0	0	0	1	6
LGH02-A-49	Methanosarcinales	Methanococcoides methylutensDSM2657T	97%	FR733669.1	0	0	0	0	0	0	0	0	0	0	0	0	1	1	0	2
LGH02-A-50	Methanosarcinales	strain NaT1	96%	Y16946.1	0	0	0	0	0	0	0	0	0	0	0	0	0	2	0	2
LGH02-A-51	Methanosarcinales	Methanococcoides methylutens DSM2657T	96%	FR733669.1	0	0	0	0	0	0	0	0	1	2	0	0	0	0	1	4
LGH02-A-52	Methanosarcinales	Methanosarcina semesiae MD1	97%	NR_028182.1	0	0	0	0	0	0	0	0	0	0	0	0	2	0	1	3
LGH02-A-53	Methanosarcinales	Methanosarcina semesiae MD1	95%	NR_028182.1	0	0	0	0	0	0	0	0	0	0	0	0	11	0	2	13
LGH02-A-60	Methanosarcinales	Methanococcoides methylutensDSM2657T	97%	FR733669.1	0	0	0	0	0	0	0	0	0	0	0	0	1	0	0	1
LGH02-A-61	Methanosarcinales	Methanolobus profundi MobM	98%	NR_041665.1	0	0	0	0	0	0	0	0	0	0	0	0	1	0	0	1
LGH02-A-63	Methanosarcinales	Methanococcoides sp. NM1	95%	HE862408.1	0	0	0	0	0	0	0	0	1	0	0	0	0	0	0	1
LGH02-A-04	Methanosarcinales	Methanosarcina semesiae MD1	98%	NR_028182.1	7	1	10	16	0	7	12	0	0	0	0	0	0	10	0	63
LGH02-A-16	Methanosarcinales	Methanococcoides sp. strain NaT1	96%	Y16946.1	0	1	0	1	0	0	0	0	0	0	0	0	0	0	0	2
LGH02-A-08	Methanosarcinales	Methanosarcina semesiae MD1	98%	NR_028182.1	0	0	0	0	0	0	1	0	0	0	0	0	0	0	0	1
LGH02-A-03	Methanosarcinales	Methanococcoides sp. strain NaT1	95%	Y16946.1	0	0	1	0	0	0	0	0	0	0	0	0	0	0	0	1
LGH02-A-09	Methanosarcinales	Methanosarcina semesiae MD1	94%	NR_028182.1	0	0	1	0	0	0	0	0	0	0	0	0	0	0	0	1
LGH02-A-70	SAGMEG	Uncultured archaeon SAGMA-Q	94%	AB050222.1	0	0	0	0	0	0	0	0	0	0	0	0	0	0	1	1
LGH02-A-37	SAGMEG	Uncultured Thermoplasmatales archaeon clone GN210D12A	95%	EU731600.1	1	0	0	0	0	0	0	0	0	0	0	0	0	0	0	1
LGH02-A-36	SCG	Uncultured crenarchaeote clone HAuD-MA29	98%	AB113626.1	2	1	0	0	0	0	0	0	0	0	0	0	0	1	0	4
Sum					97	71	74	71	72	74	89	66	67	52	55	53	58	107	67	1073
